# The dual GGDEF/EAL domain enzyme PA0285 is a *Pseudomonas* species housekeeping phosphodiesterase regulating early attachment and biofilm architecture

**DOI:** 10.1016/j.jbc.2024.105659

**Published:** 2024-01-16

**Authors:** Kira Eilers, Joey Kuok Hoong Yam, Xianghui Liu, Yu Fen Goh, Ka-Ning To, Patricia Paracuellos, Richard Morton, Jaime Brizuela, Adeline Mei Hui Yong, Michael Givskov, Sven-Andreas Freibert, Gert Bange, Scott A. Rice, Wieland Steinchen, Alain Filloux

**Affiliations:** 1 CBRB Centre for Bacterial Resistance Biology, Department of Life Sciences, Imperial College London, London, United Kingdom; 2 Singapore Centre for Environmental Life Sciences Engineering, Nanyang Technological University, Singapore; 3 Costerton Biofilm Center, Department of Immunology and Microbiology, University of Copenhagen, Denmark; 4 Philipps University Marburg, Center for Synthetic Microbiology (SYNMIKRO), Marburg, Germany; 5 Microbiomes for One Systems Health and Agriculture and Food, CSIRO, Westmead, New South Wales, Australia

**Keywords:** *Pseudomonas*, c-di-GMP, phosphodiesterase, diguanylate cyclase, biofilm

## Abstract

Bacterial lifestyles depend on conditions encountered during colonization. The transition between planktonic and biofilm growth is dependent on the intracellular second messenger c-di-GMP. High c-di-GMP levels driven by diguanylate cyclases (DGCs) activity favor biofilm formation, while low levels were maintained by phosphodiesterases (PDE) encourage planktonic lifestyle. The activity of these enzymes can be modulated by stimuli-sensing domains such as Per-ARNT-Sim (PAS). In *Pseudomonas aeruginosa,* more than 40 PDE/DGC are involved in c-di-GMP homeostasis, including 16 dual proteins possessing both canonical DGC and PDE motifs, that is, GGDEF and EAL, respectively. It was reported that deletion of the EAL/GGDEF dual enzyme PA0285, one of five c-di-GMP–related enzymes conserved across all *Pseudomonas* species, impacts biofilms. PA0285 is anchored in the membrane and carries two PAS domains. Here, we confirm that its role is conserved in various *P. aeruginosa* strains and in *Pseudomonas putida*. Deletion of PA0285 impacts the early stage of colonization, and RNA-seq analysis suggests that expression of *cupA* fimbrial genes is involved. We demonstrate that the C-terminal portion of PA0285 encompassing the GGDEF and EAL domains binds GTP and c-di-GMP, respectively, but only exhibits PDE activity *in vitro*. However, both GGDEF and EAL domains are important for PA0285 PDE activity *in vivo*. Complementation of the PA0285 mutant strain with a copy of the gene encoding the C-terminal GGDEF/EAL portion *in trans* was not as effective as complementation with the full-length gene. This suggests the N-terminal transmembrane and PAS domains influence the PDE activity *in vivo*, through modulating the protein conformation.

Bacteria rely on complex regulatory cascades to adapt to their ever-changing environment (1). *Pseudomonas aeruginosa*, a ubiquitous Gram-negative bacterium capable of opportunistic infection, can multiply in a variety of water and soil-based environments, as well as human, animal, or plant hosts (2). It is equipped with numerous virulence factors including secreted proteins such as alkaline protease (3) or elastase, which are both involved in *P. aeruginosa* infections (4) and can compromise the integrity of host epithelia (5). Within an infected host, *P. aeruginosa* causes both acute and chronic infections, the latter of which is associated with a poor outcome in bronchiectasis patients, particularly those with cystic fibrosis (6, 7, 8). The ability to thrive in different biological niches and to switch between acute and chronic infectious life cycles, results from the large genome of *P. aeruginosa* (approximately six Mbp), eight percent of which encodes for genes involved in regulatory cascades (9).

A regulatory network of special prominence in *P. aeruginosa* is represented by signaling systems utilizing the c-di-GMP second messenger, which effectively controls phenotypes such as attachment (Cup fimbriae, pili, flagellum), type IV pili regulation, biofilm formation and dispersal, exopolysaccharide production, virulence, type II secretion (T2SS), which is broadly conserved in *Pseudomonas* species (10), and motility (swimming, twitching, swarming) (11, 12). Forty one genes in *P. aeruginosa* code for c-di-GMP metabolizing enzymes of very diverse domain topologies that likely serve to integrate various environmental signals (13, 14). The cellular concentration of c-di-GMP is controlled by the antagonistic enzymatic activities of two protein families. Diguanylate cyclases (DGCs) synthesize c-di-GMP from two molecules of GTP and are characterized by the presence of a GGDEF motif in their catalytic center (15). Phosphodiesterases (PDEs) may either contain an EAL or HD-GYP catalytic motif and facilitate the hydrolysis of c-di-GMP to the linear diguanylate pGpG or even further to GMP (16, 17).

A special group of c-di-GMP metabolizing enzymes is characterized by the presence of both canonical catalytic domains, that is, GGDEF and EAL. A GGDEF/EAL tandem architecture is found in 16 of the 41 c-di-GMP encoding genes in the *P. aeruginosa* PAO1 reference strain (13). These GGDEF/EAL dual domain-containing proteins are coupled to a variety of additional sensor and/or regulatory domains, including GAF, PBPb, MASE1, CHASE4, and most frequently, Per-ARNT-Sim (PAS) domains. These presumably recognize chemical and physical changes in the extracellular and intracellular conditions by sensing cues directly or indirectly *via* the binding of small molecules, protein–protein interactions, and temperature, to regulate the antagonistic DGC and PDE activities (18, 19, 20). There are several possible activities that dual GGDEF/EAL proteins can adopt: (i) inactivation of both the GGDEF and EAL domain, resulting in no DGC or PDE activity but maintained c-di-GMP binding ability; (ii) inactive GGDEF motif but intact EAL domain, resulting in PDE activity; (iii) inactive EAL motif but intact GGDEF domain, resulting in DGC activity; and (iv) both domains are intact resulting in bifunctional DGC and PDE activities. Some of the GGDEF/EAL dual domain-containing proteins, for example, BifA (21, 22), DipA (23), NbdA (24), PA2567 (25), and PA5295/ProE (26), have been experimentally shown to exhibit PDE activity, effectively degrading c-di-GMP and decreasing cellular c-di-GMP contents (27). RmcA (28) and RbdA (29) accommodate both intact GGDEF and EAL domains but predominantly behave as PDEs showing no GGDEF-mediated c-di-GMP synthesis *in vitro.* In contrast, MucR (30) and MorA (31) also carry two intact domains but have been shown to be bifunctional enzymes with both DGC and PDE activities *in vitro*, yet adopt primarily DGC (MucR) or PDE (MorA) activity *in vivo* under tested experimental conditions.

In our previous phenotypic screening of the 41 c-di-GMP–metabolizing enzyme genes, we revealed the GGDEF/EAL-containing protein PA0285 to have one of the most pronounced effect on biofilm and attachment phenotypes, as well as on intracellular c-di-GMP levels (13). This was confirmed in a parallel study that further suggested that PA0285, also named PipA, positively controlled the Pf4 bacteriophage gene cluster, implicating a role between phage production and autoaggregation (32). In the present study, we further characterize PA0285 and demonstrate that it exhibits PDE activity but no DGC activity *in vitro*, whereas GTP and c-di-GMP substrates bind to their respective GGDEF and EAL domains. Nevertheless, site-directed mutagenesis of the GGDEF motif impedes PDE activity of PA0285 *in vitro* and *in vivo,* suggesting an interdomain communication. The functional impact of PA0285 on the early attachment during biofilm formation is conserved among *Pseudomonas* strains and RNA-seq analysis of *P. aeruginosa* PAO1 suggests that PA0285 alters the transcription of biofilm-related genes, such as the fimbrial *cupA* genes that have been proposed to be involved in the early attachment (33).

## Results

### PA0285 influence on biofilm formation is conserved in *Pseudomonas* species

**Figure S1:**
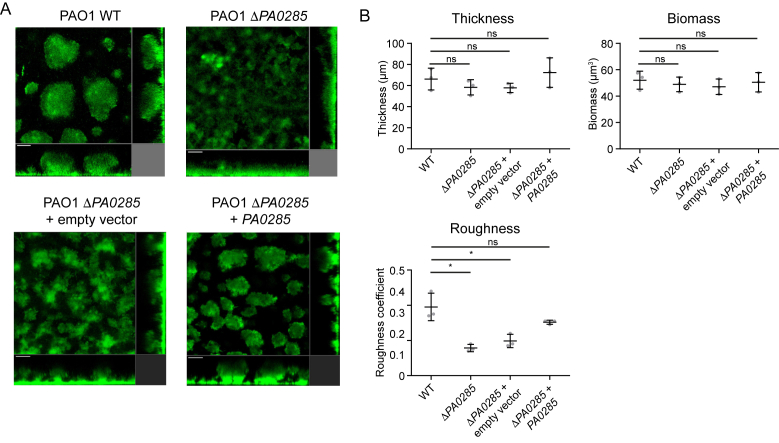
**Biofilm characteristics of PAO1 mutant strains.***A*, micrographs of *P. aeruginosa* PAO1 WT, the *PA0285* deletion and *PA0285* deletion strains complemented with either empty vector or *PA0285*. Scale bar, 50 μm. GFP-tagged PAO1 WT and mutant strains were grown for 72 h in biofilm flow cell chambers under continuous feeding of ABTG medium. Confocal scanning laser microscopy images were taken (three channels: seven images per channel). A representative image for each strain is shown. *B*, thickness, biomass and roughness coefficient (indicator of biofilm heterogeneity) of *P. aeruginosa* PAO1 and mutant strain from *A* were analyzed using biofilm quantification program COMSTAT. Data represent mean ± SD of three biological replicates. Unpaired two-tailed t-tests (with Welch’s correction) were used to compare the mutant strains with WT. *Asterisks* indicate *p*-values: ∗*p* ≤ 0.05; ns, not significant. Determined *p*-values are: thickness: 0.3478 (WT *vs.* Δ*PA0285*), 0.2944 (WT *vs.* Δ*PA0285* + empty vector), 0.5767 (WT *vs.* Δ*PA0285* + *PA0285*); biomass: 0.5718 (WT *vs.* Δ*PA0285*), 0.3950 (WT *vs.* Δ*PA0285* + empty vector), 0.8017 (WT *vs.* Δ*PA0285* + *PA0285*); roughness coefficient: 0.0286 (WT *vs.* Δ*PA0285*), 0.0329 (WT *vs.* Δ*PA0285* + empty vector), 0.1923 (WT *vs.* Δ*PA0285* + *PA0285*).

**Figure S2:**
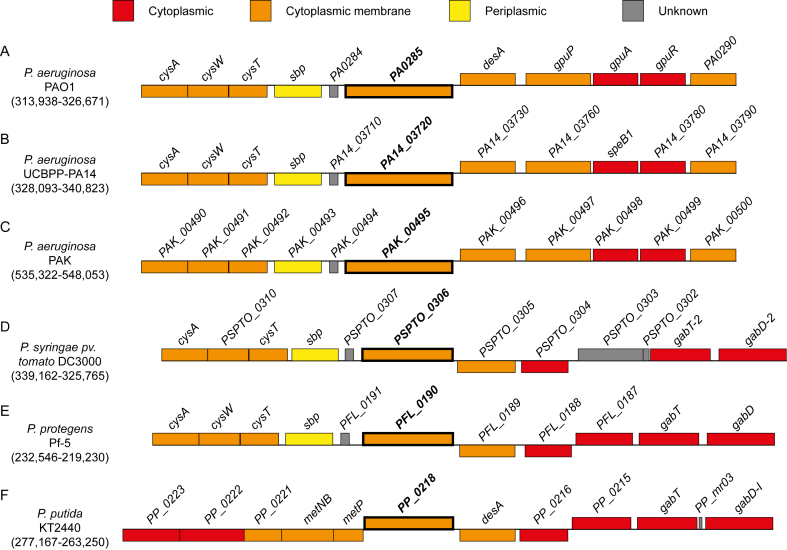
**Conservation of *PA0285* orthologs among *Pseudomonas* species.***A*–*F*, neighboring genes of *PA0285* orthologs in *A, P. aeruginosa* PAO1, *B*, *P. aeruginosa* UCBPP-PA14, *C*, *P. aeruginosa* PAK, *D*, *P. syringae pv. tomato* DC3000, *E*, *P. protegens* Pf-5, and *F*, *P. putida* KT2440 retrieved from the *Pseudomonas* Genome Database (73). Numbers in parenthesis denote the base pairs of the displayed genome sections. Note that in *P. syringae pv. tomato* DC3000, *P. protegens* Pf-5 and *P. putida* KT2440 the *PA0285* ortholog-enframing genes are inverted. Note that the orientation was inverted in panels *D*–*F* for better comparability.

PA0285 is one of five broadly conserved c-di-GMP–metabolizing enzymes in *Pseudomonas* species, along with PA0290, PA4367, PA5017, and PA5487 (34). Among these, PA0290 and PA5487 (DgcH) were predicted as DGCs, while PA0285, PA4367 (BifA), and PA5017 (DipA) are dual GGDEF/EAL domain-containing enzymes. In our previous work using *P. aeruginosa* PAO1, a Δ*PA0285* mutant strain had the highest increase in c-di-GMP levels and initial surface attachment (13) and likewise modulated the architecture of mature biofilms (Fig. S1). PA0285 and its genetic location are conserved in most *Pseudomonas* strains (34). It is found downstream of a gene cluster, *cysAWT-sbp*, involved in sulfur transport and metabolism, and upstream of *desA*, encoding a fatty acid desaturase in *P. aeruginosa* PAO1, PA14, and PAK strains (Fig. S2). This organization is, though inverted, also present in *Pseudomonas syringae pv. tomato* DC3000 and *Pseudomonas protegens* Pf-5. In *Pseudomonas putida* KT2440 the PAO1 PA0285 ortholog, PP_0218, is between *desA* and *metP*/*metNB*/PP_0221 and annotated as components of a methionine ABC transporter (Fig. S2).

**Figure 1:**
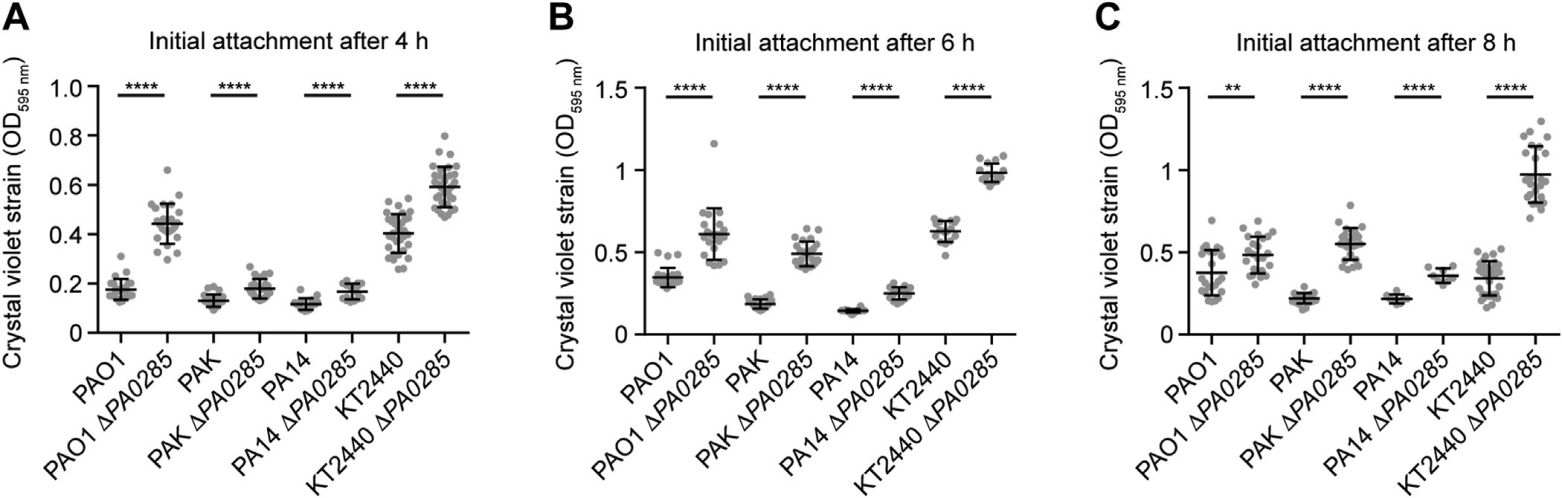
**Influence of PA0285 on biofilm formation is conserved in *Pseudomonas* species.***A*–*C*, deletion of the *PA0285* ortholog in *P. aeruginoa* strains PAO1, PAK, and PA14, and *Pseudomonas putida* KT2440 promotes initial surface attachment reflected by *crystal violet staining*, assayed after (*A*) 4 h, (*B*) 6 h, and (*C*) 8 h cultivation in LB medium, by a microtiter plate assay. Data represent mean ± SD of eight technical replicates from three biological replicates each. Unpaired two-tailed *t* tests (with Welch’s correction) were used to compare the WT and Δ*PA0285* ortholog mutant strains. *Asterisks* indicate *p* values: ∗∗*p* ≤ 0.01, ∗∗∗∗*p* ≤ 0.0001. Determined *p* values are: PAO1: <0.0001 (4 h), <0.0001 (6 h), 0.0047 (8 h), PAK: <0.0001 (4 h), <0.0001 (6 h), <0.0001 (8 h), PA14: <0.0001 (4 h), <0.0001 (6 h), <0.0001 (8 h), KT2440: <0.0001 (4 h), <0.0001 (6 h), <0.0001 (8 h). LB, lysogeny broth.

To elucidate whether the role of PA0285 would be conserved across *Pseudomonas* strains and species, we probed the impact of *PA0285* deletions (Δ*PA0285*) in PAO1, PA14, PAK, or KT2440 (Fig. 1). In previous studies, we reported that a PAO1ΔPA0285 mutant had an impact on surface attachment after 6 h of growth in lysogeny broth (LB) medium in a microtiter plate assay (13). Here, we consolidated this result and further demonstrated that the *PA0285* mutation has a clearer impact at an earlier time of attachment, notably 4 h, while after 8 h the difference between the WT PAO1 and PAO1Δ*PA0285* mutant is less significant. For *P. putida* KT2440, the largest difference in attachment was observed after 8 h, while the difference was minimal after 4 h. Similarly, for *P. aeruginosa* PAK and PA14, the impact was more visible after 6 and 8 h of growth, respectively (Fig. 1). We thus conclude that PA0285 has a conserved impact on attachment, albeit with a variable strain-dependent influence.

### Both GGDEF and EAL activity are important for PA0285 function *in vivo*

**Figure 2:**
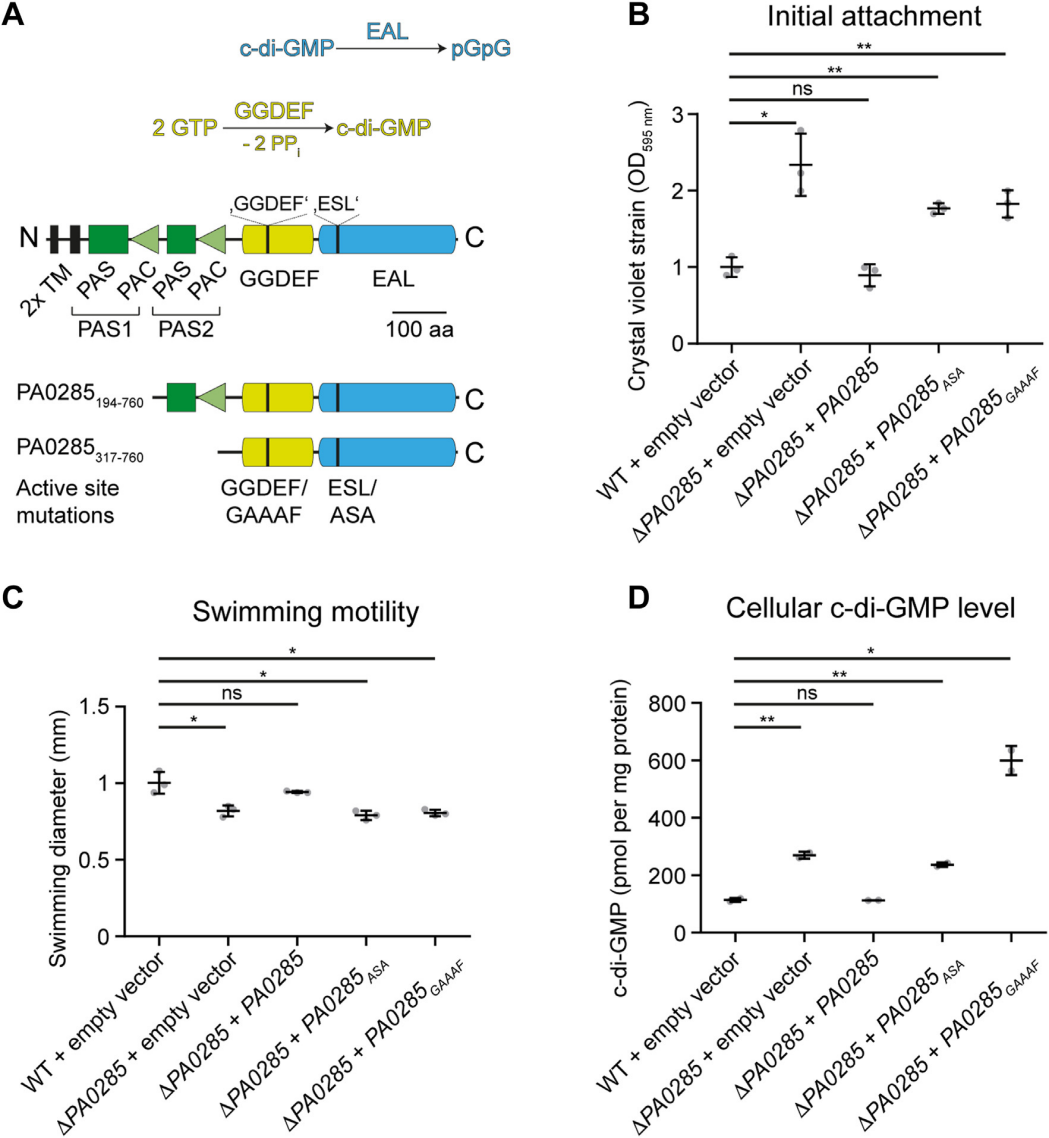
**The GGDEF and EAL activities are required for PA0285 function *in vivo*.***A*, schematic depiction of PA0285 domain topology. Domain annotations are per UniProt-ID Q9I6K5. The boundaries of PA0285 constructs are indicated below. *B*–*D*, impact of PA0285 on (*B*) initial attachment, (*C*) swimming motility, and (*D*) intracellular c-di-GMP levels of *Pseudomonas aeruginosa* PAO1. Data were normalized to WT + empty vector and represent mean ± SD of three biological replicates (in *B* and *C*) or nonnormalized representing mean ± SD of two biological replicates (in *D*). Unpaired two-tailed *t* tests (with Welch’s correction) were used to compare WT *versus* mutant strains. *Asterisks* indicate *p* values: ∗*p* ≤ 0.05, ∗∗*p* ≤ 0.01; ns, not significant. Determined *p* values are in *B*: 0.0212 (Δ*PA0285* + empty vector), 0.3969 (Δ*PA0285* + *PA0285*), 0.0027 (Δ*PA0285* + *PA0285*_*ASA*_), 0.0038 (Δ*PA0285* + *PA0285*_*GAAAF*_); in *C*: 0.0287 (Δ*PA0285* + empty vector), 0.2802 (Δ*PA0285* + *PA0285*), 0.0220 (Δ*PA0285* + *PA0285*_*ASA*_), 0.0324 (Δ*PA0285* + *PA0285*_*GAAAF*_); in *D*: 0.0092 (Δ*PA0285* + empty vector), 0.8146 (Δ*PA0285* + *PA0285*), 0.0038 (Δ*PA0285* + *PA0285*_*ASA*_), 0.0427 (Δ*PA0285* + *PA0285*_*GAAAF*_).

PA0285 is a 760 amino acid protein that contains the GGDEF and EAL domains within its C-terminal portion (Fig. 2*A*). The former harbors the eponymous “GGDEF” amino acid motif (residues 409-414) required for DGC activity; however a DGC inhibitory site (I-site) located upstream of the GGDEF active site in some DGC and GGDEF/EAL enzymes, for example, RbdA and RmcA (28, 29), is not present in PA0285. It should be noted that an “ESL” motif present in PA0285 (residues 537-539) replaces the canonical “EAL” found in the catalytic center of most PDE enzymes (Fig. 2*A*). Further, PA0285 contains an intact loop 6 (residues 658-666), the presence of which is associated with productive PDE activity (25, 35). The PA0285 N-terminal portion encompasses two transmembrane portions that delineate a short predicted periplasmic region, and two cytoplasmic PAS domains, which often serve as signal perceiving or transducing entities (*e.g.*, cofactor binding or oligomer formation (36)).

Characterization of the *P. aeruginosa* PAO1Δ*PA0285* mutant showed enhanced initial attachment (Fig. 2*B*) and a reduced swimming diameter on motility-promoting agar plates (Fig. 2*C*) that are linked to elevated c-di-GMP levels in the mutant strain compared to the WT (Fig. 2*D*). This is in line with the prevalent notion that elevated c-di-GMP promotes a sessile lifestyle (17, 37). Both phenotypes can be complemented in the presence of IPTG by introduction of the entire *PA0285* gene under control of the *lac* promoter, concomitant with normalization of c-di-GMP levels (Fig. 2, *B*–*D*). However, complementation of PAO1Δ*PA0285 in trans* with *PA0285* mutant genes in which the DGC or PDE activities are disrupted by variation of the catalytic GGDEF and EAL domain motifs (GGDEF/GAAAF and ESL/ASA, respectively) did not restore the WT phenotypes (Fig. 2, *B* and *C*). Furthermore, the elevated c-di-GMP levels of the Δ*PA0285* mutant strain were not restored to lower WT levels by expression of either *PA0285* GGDEF/GAAAF or EAL/ASA alleles, in contrast to complementation with native PA0285 (Fig. 2*D*). This strongly suggests that PA0285 primarily acts as a PDE but that both GGDEF and EAL domain activities are required for PA0285 function.

### The GGDEF/EAL tandem domains of PA0285 show PDE but no DGC activity *in vitro*

**Figure 3:**
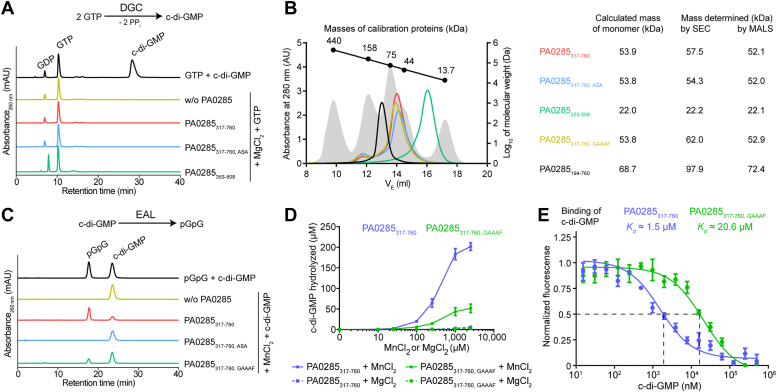
**The GGDEF/EAL tandem domains of PA0285**_**317-760**_**show PDE but lack DGC activity *in vitro*.***A*, representative UV traces of PA0285 enzymatic reactions probing DGC activity in presence of GTP substrate and Mg^2+^ ion cofactor. Reactions contained (where indicated) 10 μM PA0285 (PA0285_317-760_, PA0285_317-760, ASA_ or PA0285_353-508_), 2.5 mM GTP, and 2.5 mM MgCl_2_, and were incubated for 60 min at 37 °C prior quenching and analysis of nucleotide content by HPLC analysis. A mixture of GTP and c-di-GMP (250 μM each) served as standard for identification based on retention time. PA0285_353-508_ showed elevated GDP probably attributable to an unknown GTPase contaminant in our preparations of this particular variant. *B*, the apparent molecular weight of PA0285_317-760_ (*red*), PA0285_317-760, ASA_ (*blue*), PA0285_353-508_ (*green*), PA0285_317-760, GAAAF_ (*ochre*), and PA0285_194-760_ (*black*) was determined by analytical size-exclusion chromatography (SEC) on a Superdex200 10/30 Gl column, which was calibrated (*grey shade*) with a mixture of ferritin (440 kDa), aldolase (158 kDa), conalbumin (75 kDa), ovalbumin (44 kDa), and RNase A (13.7 kDa). The molecular weight was determined either from the elution behavior on SEC alone or by multiangle light scattering (MALS) coupled after the SEC separation. *C*, representative UV traces of PA0285 enzymatic reactions probing EAL activity in presence of c-di-GMP substrate and Mn^2+^ ion cofactor. Reactions contained (where indicated) 2.5 μM PA0285 (PA0285_317-760_, PA0285_317-760, GAAAF_, or PA0285_317-760, ASA_), 250 μM c-di-GMP and 2.5 mM MnCl_2_ and were incubated for 60 min at 37 °C prior quenching and analysis of nucleotide content by HPLC analysis. A mixture of pGpG and c-di-GMP (250 μM each) served as standard for identification based on retention time. *D*, dose-dependent characterization of PA0285 EAL activity. Reactions contained 2.5 μM PA0285_317-760_ or PA0285_317-760, GAAAF_, 250 μM c-di-GMP and increasing concentrations of either MgCl_2_ or MnCl_2_, that is, 0, 10, 25, 100, 250, 1000, or 2500 μM. The concentration of hydrolyzed c-di-GMP was determined after 60 min of incubation at 37 °C by HPLC quantification of the pGpG reaction product. Data represent mean ± SD of three biological replicates (independent PA0285_317-760_ protein preparations). *E*, MST experiments probing c-di-GMP binding to PA0285_317-760_ and PA0285_317-760, GAAAF_. DGC, diguanylate cyclase; MST, microscale thermophoresis; PDE, phosphodiesterase.

**Figure S3:**
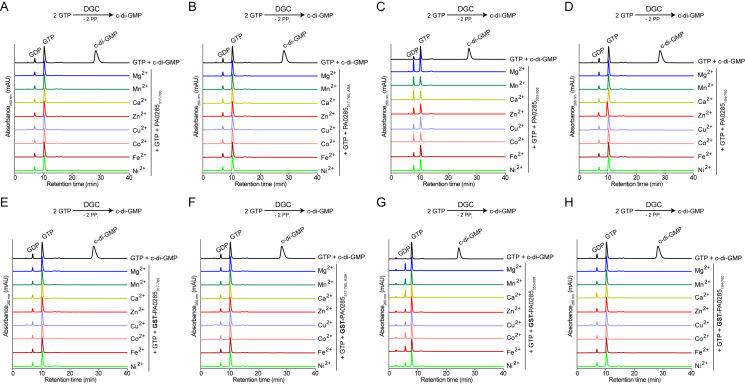
**The GGDEF/EAL tandem domains of PA0285**_**317-760**_**do not exhibit DGC activity *in vitro*.***A*–*D*, representative UV traces of enzymatic reactions probing DGC activity of 2.5 μM *A*, PA0285_317-760_, *B*, PA0285_317-760, ASA_, *C*, PA0285_353-508_, or *D*, PA0285_194-760_ in presence of 2.5 mM GTP substrate and 2.5 mM of various divalent metal ion cofactors provided as MgCl_2_, MnCl_2_, CaCl_2_, ZnCl_2_, CuCl_2_, CoCl_2_, FeSO_4_, or NiSO_4_. The reactions were allowed to proceed for 60 min at 37 °C prior quenching and analysis of nucleotide content by HPLC analysis. A mixture of GTP and c-di-GMP (250 μM each) served as standard for identification based on retention time. *Panels E*–*H* display representative UV traces of enzymatic reactions for DGC activity employing 2.5 μM of GST-tagged proteins, *i.e.*, *E*, GST-PA0285_317-760_, *G*, GST-PA0285_317-760, ASA_, *G*, GST-PA0285_353-508_, or *H*, GST-PA0285_194-760_ similarly. Note that both tagless and GST-tagged PA0285_353-508_ (*panels C* and *G*, respectively) showed elevated GDP probably attributable to an unknown GTPase contaminant in our preparations of this particular variant.

To assess the enzymatic activity of PA0285 *in vitro*, we purified a truncated PA0285 variant encompassing the GGDEF and EAL domains (PA0285_317-760_) and tested this variant for DGC and EAL activity in presence of GTP or c-di-GMP substrate, respectively, by following the reactions with HPLC. Incubation of PA0285_317-760_ together with GTP in presence of Mg^2+^ (Fig. 3*A*) or other divalent metal ions (Fig. S3*A*) did not reveal any visible c-di-GMP product, suggesting a lack of DGC activity. DGC activity was also not detected for a variant in which the ESL motif in PA0285 was replaced by ASA (PA0285_317-760, ASA_) or the isolated GGDEF domain (PA0285_353-508_), thus providing no support for the hypothesis that the EAL domain would potentially exert a negative control over activity of the neighboring GGDEF domain (Figs. 3*A* and S3, *B* and *C*).

In size-exclusion chromatography (SEC), the migration behavior of PA0285_317-760_ variants and the isolated GGDEF domain suggests a monomeric status (Fig. 3*B*), possibly explaining the lack of c-di-GMP synthesis, which relies on the formation of GGDEF domain dimers (38). Similarly, a PA0285 construct encompassing the PAS domain that directly precedes the GGDEF/EAL tandem, PA0285_194-760_, appeared as a monomer as suggested from the molecular weight determined through multiangle light scattering (MALS), although the inclusion of the PAS domain seemingly resulted in a nonglobular overall protein organization reflected by the SEC-derived molecular weight higher than that of a monomer (Fig. 3*B*).

In contrast, the formation of a pGpG product was observed when incubating PA0285_317-760_ with c-di-GMP substrate in the presence of Mn^2+^, thus demonstrating PDE activity (Fig. 3*C*). The loss of PDE activity in PA0285_317-760, ASA_ (Fig. 3, *C* and *D*) infers that PDE activity relies on the presence of an intact EAL domain active site. However, substitution of the GGDEF domain motif by GAAAF (PA0285_317-760, GAAAF_) reduced PDE activity by about 4-fold in the presence of a 2.5 mM Mn^2+^ cofactor (Fig. 3, *C* and *D*), reflecting an effect of complementation with PA0285_317-760, GAAAF_ on attachment, biofilm architecture and c-di-GMP levels observed *in vivo* (Fig. 2, *B*–*D*). Degradation of c-di-GMP to pGpG by PA0285_317-760_ was also apparent, albeit proceeding much less efficiently when employing Mg^2+^ as metal ion cofactor instead of Mn^2+^ (Fig. 3*D*). The reduced activity of PA0285_317-760, GAAAF_ compared to PA0285_317-760_ was persistent at lower concentrations of Mn^2+^, in keeping with an overall decrease in PDE activity, which is eventually reasoned in a reduced capacity of binding the c-di-GMP substrate as suggested by microscale thermophoresis (MST) experiments (Fig. 3, *D* and *E*).

Taken together, these data show that the GGDEF/EAL tandem domains of PA0285 exhibit manganese ion-dependent PDE but no DGC activity *in vitro*, and further suggest that the GGDEF domain somehow modulates activity of the EAL domain.

### Substrate binding to the PA0285 GGDEF/EAL tandem domains

To elucidate how the GGDEF domain regulates PDE activity by the EAL domain we employed a hydrogen/deuterium exchange (HDX) coupled to mass spectrometry (MS) (Dataset S1) (39, 40). HDX-MS allows for characterization of protein-ligand interfaces by analyzing the exchange of the peptide backbone amide protium for deuterium after incubation of a protein in deuterium oxide (D_2_O). Regions that are obscured by ligand interaction typically exhibit lower HDX than the protein in the apo state. We employed GTP and c-di-GMP at final concentrations of 2.5 mM and 250 μM, respectively, to promote a high portion of nucleotide-bound PA0285 in the sample based on *K*_*d*_ values for other GGDEF or EAL domain–containing proteins (38, 41, 42).

**Figure 4:**
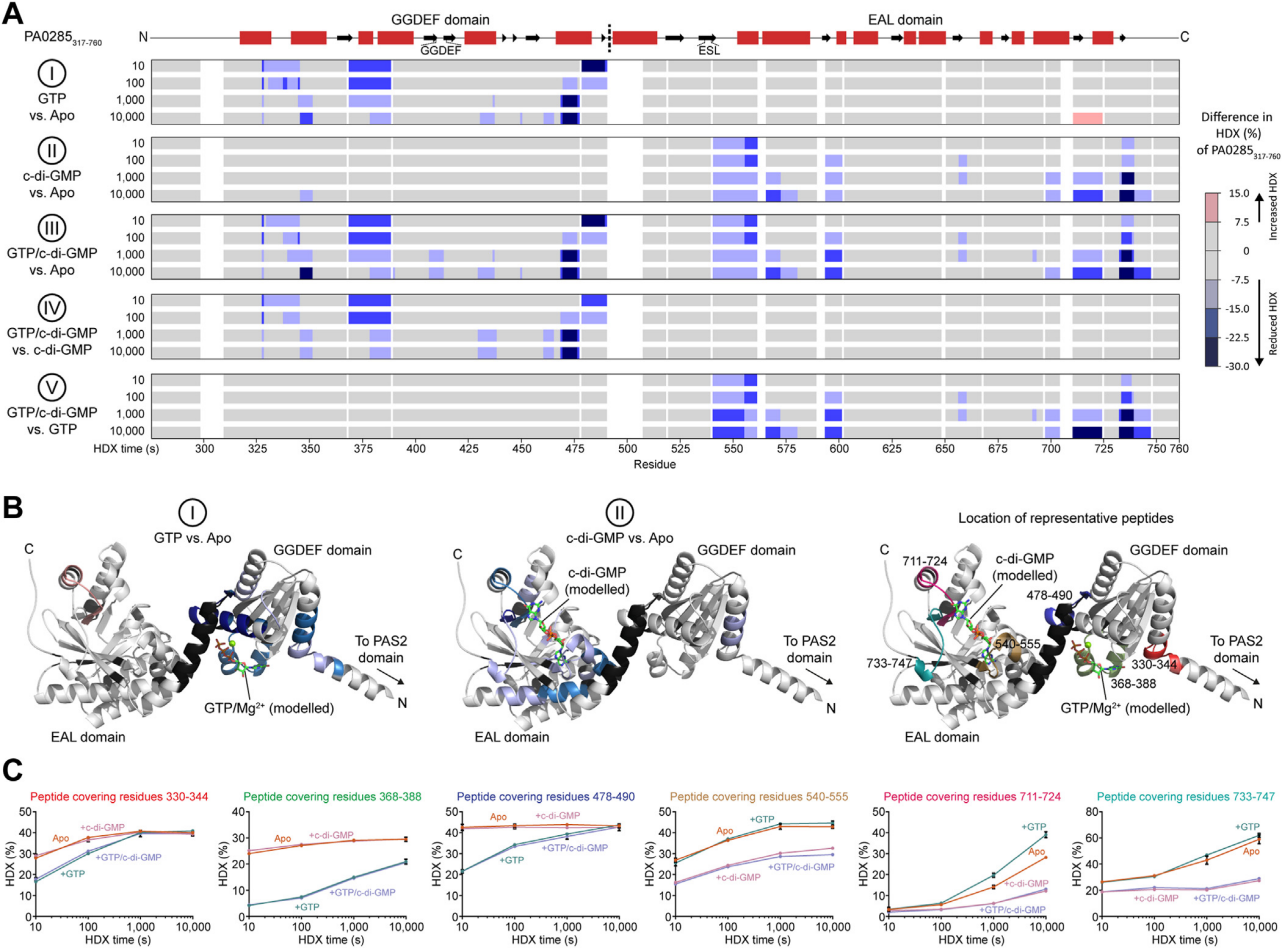
**Conformational changes of PA0285**_**317-760**_**related to binding of GTP and c-di-GMP substrates.***A*, the difference in HDX of PA0285_317-760_ depending on the presence of GTP (2.5 mM) or/and c-di-GMP (250 μM) projected on its amino acid sequence. The predicted secondary structure is indicated above (*red boxes*, α-helices; *black arrows*, β-strands). HDX is given per residue (Dataset S1). *B*, the differences in HDX induced by GTP or c-di-GMP are projected onto the AlphaFold structural model (AF-Q9I6K5-F1-model_v4; (43, 90)) of PA0285 (residues 317-760 are displayed). The approximate positions of GTP and c-di-GMP originate from superimposition with the crystal structures of GTP-bound and c-di-GMP–bound *P. aeruginosa* RbdA (PDB-identifiers 5XGD and 5XGE, respectively; (29)). If different changes in HDX over the time course were observed, the strongest difference at any time point (see Fig. 4*A*) was projected. *Black color* denotes areas for which no peptides were obtained. *C*, progression of HDX over time displayed for six selected representative peptides. Data represent mean ± SD of three technical replicates (independent HDX reactions from same protein preparation). HDX, hydrogen/deuterium exchange.

Firstly, we confirmed that both domains of PA0285_317-760_ could coordinate their respective substrate in isolation, which is GTP for the GGDEF and c-di-GMP for the EAL domain (Fig. 4, I-II). For each substrate, reduced HDX was found almost exclusively in the corresponding domains (Fig. 4, *A* and *B*). Although the GGDEF-containing loop was not affected in the presence of GTP, the neighboring helices exhibited reduced HDX, substantiating GTP binding (Fig. 4*B*). Supplementation of c-di-GMP restricted HDX in a region close to the ESL motif of PA0285 and further areas in close proximity of the predicted c-di-GMP binding site (*e.g.*, residues 540–555, 711–724 and 733–747; Fig. 4, *B* and *C*). No other c-di-GMP binding sites were identified besides the EAL domain active site, which is in keeping with the absence of an I-site motif near the canonical GGDEF domain.

**Figure S4:**
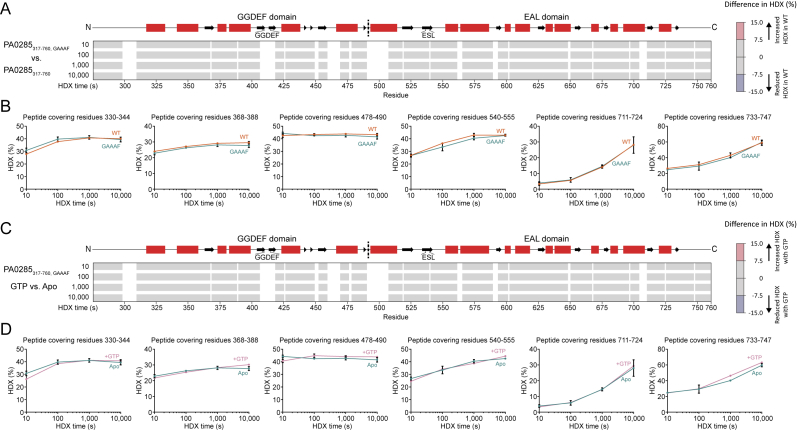
**HDX-MS analysis of the PA0285**_**317-760, GAAAF**_**variant.***A* and *C,* the difference in HDX of between *A*, PA0285_317-760, GAAAF_ and PA0285_317-760_ in the apo states, and *C*, PA0285_317-760, GAAAF_ in presence of GTP versus apo state, projected on the amino acid sequence. The predicted secondary structure is indicated above both plots (*red boxes*, α-helices; *black arrows*, β-strands). HDX is given per residue (Dataset S1). *B* and *D*, progression of HDX over time displayed for six selected representative peptides for *B*, the apo states of PA0285_317-760, GAAAF_ and PA0285_317-760_, and *D*, PA0285_317-760, GAAAF_ in presence of GTP versus apo state. Data represent mean ± SD of three technical replicates (independent HDX reactions).

Secondly, we probed the effects of combined GTP and c-di-GMP addition (Fig. S4, III-V). Here, the changes in HDX in both domains were very similar in magnitude as those evoked individually by the ligands (Fig. 4, *A* and *C*), thus suggesting that GTP does not interfere with c-di-GMP binding and vice versa.

Finally, we assessed the behavior of the PA0285_317-760, GAAAF_ variant by HDX-MS (Fig. S4). Comparison of the HDX profiles of PA0285_317-760, GAAAF_ and PA0285_317-760_ did not reveal obvious differences in their conformation (Fig. S4, *A* and *B*). The inability of PA0285_317-760, GAAAF_ to coordinate GTP, though, is evidenced from the lack of HDX reduction at its GGDEF domain (Fig. S4, *C* and *D*). This suggests that the different structure and/or conformation of PA0285_317-760, GAAAF_ and PA0285_317-760,_ which underlies the differences in PDE activity (Fig. 3, *C*–*E*), is either too small to be observed by HDX-MS or occurs in a part of the protein not assessed in our experiment, for example, residues 491 to 506, which bridge the GGDEF and EAL domains of the helix.

### GTP impedes EAL domain activity *in vitro* by scavenging Mn^2+^, independently of the GGDEF domain

**Figure S5:**
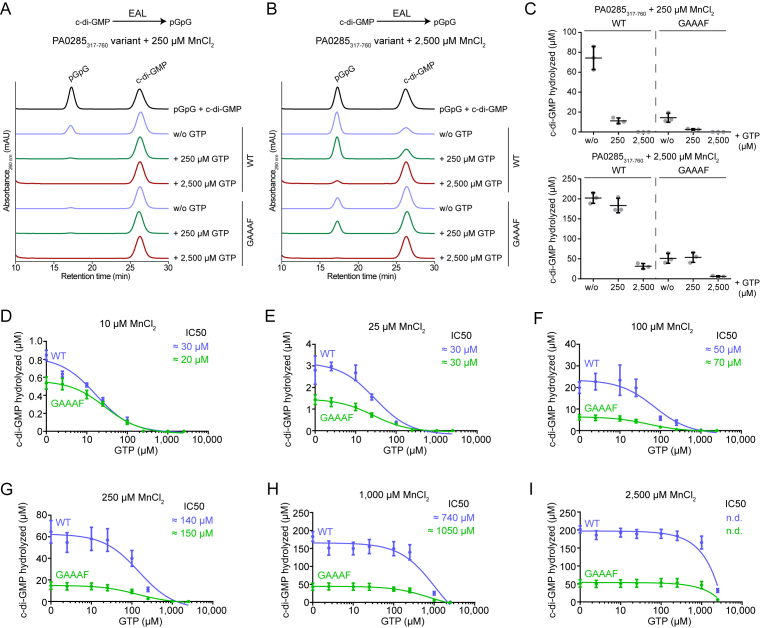
**Mn**^**2+**^**scavenging by GTP impedes PA0285 PDE activity *in vitro*.***A* and *B*, Representative UV traces of enzymatic reactions containing 2.5 μM of A. PA0285_317-760_, or *B*, PA0285_317-760, GAAAF_, probing EAL activity in presence of c-di-GMP substrate, 250 or 2,500 μM Mn^2+^ ion cofactor and GTP at 0, 100 or 1,000 μM final concentration. The reactions were incubated for 60 min at 37 °C prior quenching and analysis of nucleotide content by HPLC analysis. A mixture of pGpG and c-di-GMP (250 μM each) served as standard for identification based on retention time. *C*, quantification of PDE activity of reactions shown in *A* and *B*. Data represent mean ± SD of three biological replicates (independent protein preparations). *D*–*I*. IC_50_ values for inhibition of PA0285_317-760_ (*blue*) and PA0285_317-760, GAAAF_ (*green*) PDE activity by GTP were obtained from a fit of the enzymatic activity versus the log_10_ of GTP concentration. Reactions contained 2.5 μM PA0285, 250 μM c-di-GMP, MnCl_2_ at 10, 25, 100, 250, 1,000 or 2,500 μM, and GTP was supplemented at 0, 2.5, 10, 25, 100, 250, 1,000 or 2,500 μM final concentration. Assays were allowed to proceed for 60 min at 37 °C prior quenching and HPLC analysis. Data represent mean ± SD of three biological replicates (independent protein preparations).

GGDEF’s inability to bind GTP upon variation to GAAAF could explain the impact of the GGDEF domain on EAL domain activity. We thus spiked enzymatic reactions containing PA0285_317-760_ or PA0285_317-760, GAAAF_ with GTP to assess the effect on c-di-GMP hydrolysis (Fig. S5). Reduced PDE activity of PA0285_317-760_ was evident with increasing concentration of GTP present in the reactions (Fig. S5, *A* and *C*), however, a similar trend was apparent for PA0285_317-760, GAAAF_ (Fig. S5, *A*–*C*). Titration experiments at different concentrations of PDE activity–promoting MnCl_2_ and inhibiting GTP showed a similar correlation for PA0285_317-760_ and PA0285_317-760, GAAAF,_ with very similar GTP IC_50_ values that likewise correlated with MnCl_2_ concentration (Fig. S5, *C*–*I*). Given that PA0285_317-760, GAAAF_ does not bind GTP (Fig. S4, *C* and *D*), this suggests that GTP diminishes PA0285_317-760_ PDE activity by scavenging Mn^2+^, the impact of which is alleviated by the increasing concentration of the cation.

### PA0285 is a dual GGDEF/EAL enzyme containing two PAS signaling domains

**Figure S6:**
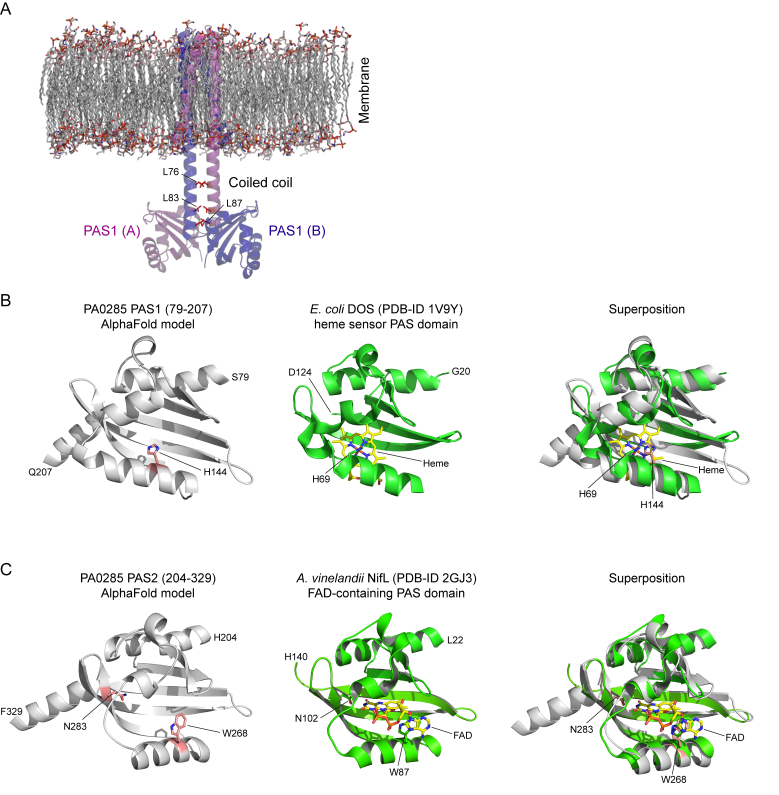
**Potential dimerization and ligand binding by the PA0285 PAS domains.***A*, AlphaFold-generated model (43) of the PA0285 residues 1-196 (TM and PAS1 domains) is shown inserted into a simplified lipid bilayer. Hydrophobic residues potentially involved in dimerization are indicated as red sticks. *B*, AlphaFold structural model of the first PAS domain (residues 79-207) of PA0285 (AF-Q9I6K5-F1-model_v4 (43)), the crystal structure of the heme PAS sensor domain (residues 20-132) from *Escherichia coli* DOS (PDB-ID 1V9Y (49) and their superposition. The heme-ligated histidine residue of *E. coli* DOS and its potential counterpart in PA0285 PAS1 are indicated. *C*, AlphaFold structural model of the second PAS domain (residues 204-329) of PA0285 (AF-Q9I6K5-F1-model_v4 (43, 72), the crystal structure of the FAD-containing redox sensor PAS domain (residues 22-140) from *Azotobacter vinelandii* NifL (PDB-ID 2GJ3 (47) and their superposition. The tryptophane and asparagine residues conferring FAD coordination by *A. vinelandii* NifL and their counterparts in PA0285 are indicated.

As reported above there is no evidence of DGC activity in the PA0285_317-760_ construct containing the GGDEF and EAL domains. It is possible that the two PAS domains identified in the N-terminal region of PA0285 (Fig. 2*A*) are required for the overall contribution in controlling the GGDEF domain activity. PAS domains are accessory domains that initiate and regulate the dimerization necessary for GGDEF domain activity (38). Modeling of the TM-PAS1 entity with AlphaFold (43) raises the possibility that PA0285 dimerization could rely on the formation of a coiled coil involving leucine residues 76, 83, and 87 (Fig. S6*A*).

**Figure 5:**
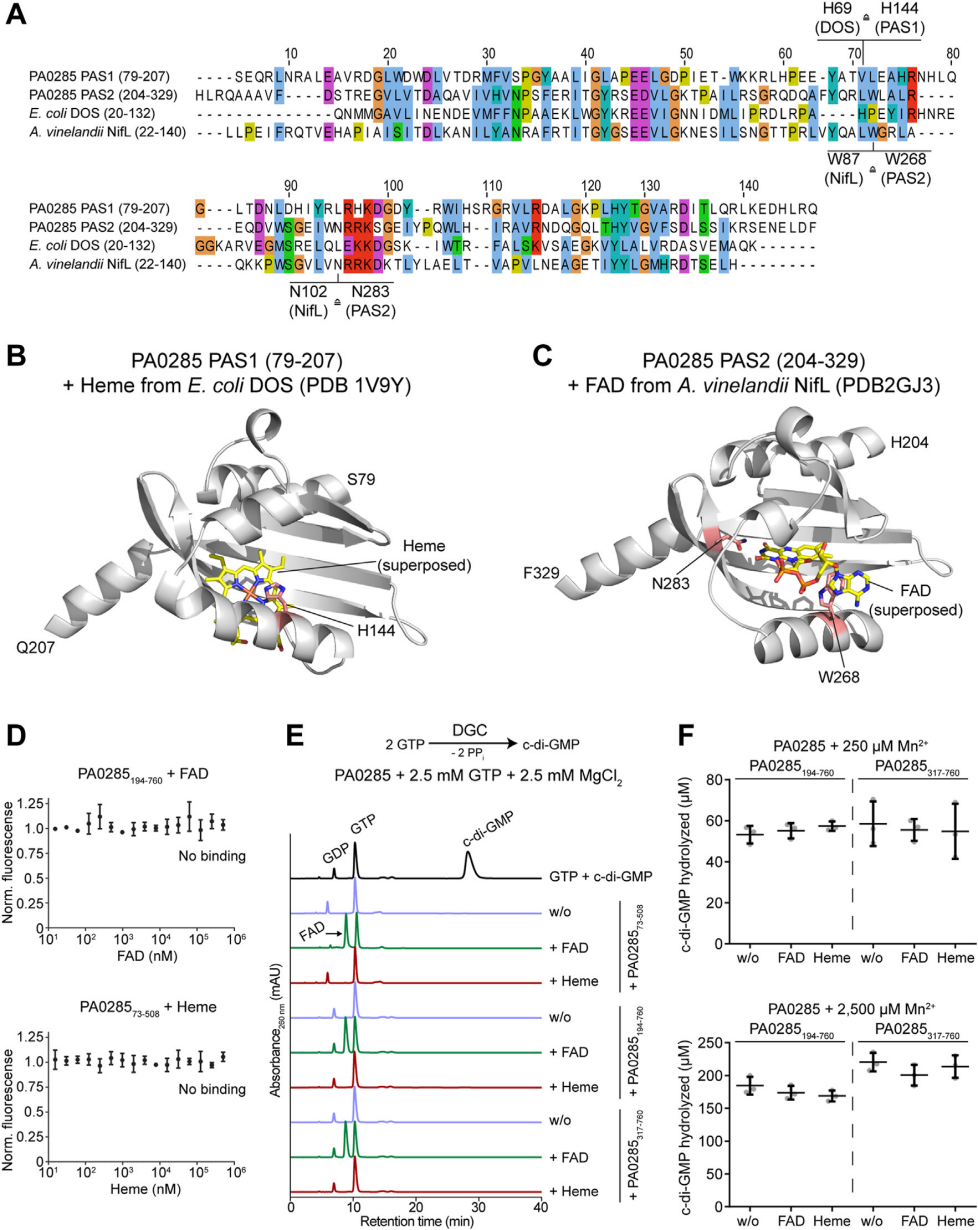
**Potential heme and FAD-binding by the PA0285 PAS domains.***A*, amino acid sequence alignment of the PA0285 first (residues 79-207) and second (residues 204-329) PAS domains of PA0285 with the heme-binding PAS domain of *Escherichia coli* DOS (UniProt-ID P76129, residues 20-132) and FAD-binding PAS domain of *Azotobacter vinelandii* NifL (UniProt-ID P30663, residues 20–140). Amino acid residues are colored according to the Clustalx scheme. *B* and *C*, AlphaFold structural model (AF-Q9I6K5-F1-model_v4; (43, 90)) of the (*B*) PAS1 domain (residues 79-207), and (*C*) PAS2 domain (residues 204-329) of PA0285. Positions of the heme and FAD cofactors (*yellow sticks*) were approximated through superposition with PAS sensor domain (residues 20-132) of *E. coli* DOS (PDB-ID 1V9Y; (49)) and the crystal structure of the FAD-containing redox sensor PAS domain (residues 22-140) from *A. vinelandii* NifL (PDB-ID 2GJ3 (47)), respectively. Residues potentially conferring coordination of the cofactors are colored in salmon and shown as *sticks*. *D*, MST experiments probing the binding of FAD to PA0285_194-760_ and heme to PA0285_73-508_. *E*, representative UV traces of PA0285 enzymatic reactions probing DGC activity in presence of GTP substrate and Mg^2+^ ion cofactor. Reactions contained PA0285_317-760_, PA0285_194-760_, or PA0285_73-508_, 2.5 mM GTP and 2.5 mM MgCl_2_, and 1 mM FAD or heme (where indicated) and were incubated for 60 min at 37 °C prior quenching and analysis of nucleotide content by HPLC analysis. *F*, PDE activity of PA0285_317-760_ or PA0285_194-760_ (2.5 μM) supplemented with either 250 or 2.500 μM MnCl_2_ and 250 μM c-di-GMP as substrate. Reactions were incubated for 60 min at 37 °C prior quenching and analysis by HPLC analysis. Data represent mean ± SD of three biological replicates (independent PA0285 protein preparations). DGC, diguanylate cyclase; FAD, flavine adenine dinucleotide; MST, microscale thermophoresis; PAS, Per-ARNT-Sim; PDE, phosphodiesterase.

PAS domains typically show low sequence identities among each other (often below 20%) but exhibit conserved three-dimensional structures (44). The PAS domain superfamily comprises different subcategories (44), and a sequence homology search of the first N-terminal PAS domain of PA0285 matched the subfamilies PAS3 (IPR013655), while the second PAS domain is not classified into a specific subgroup. Many PAS domains have been reported to bind to prosthetic groups like heme (45, 46) and flavine adenine dinucleotide (FAD) (47) *via* conserved residues, allowing perception of signals such as oxygen availability or redox potential (48, 49, 50). None of the residues required for heme coordination (51) appear to be present in either of the PA0285 PAS domains (Fig. 5*A*). However, the AlphaFold (43) model of the first PAS domain predicts a histidine residue in close proximity of the anticipated heme coordination site (Figs. 5*B* and S6*B*). For the second PAS domain, amino acid sequence alignment and superposition of the PA0285 AlphaFold model with the crystal structure of a FAD-bound PAS domain of *Azotobacter vinelandii* NifL (PDB-ID 2GJ3 (47)) renders binding of FAD by conserved tryptophane and asparagine residues plausible (Figs. 5, *A* and *C* and S6*C*).

**Figure S7:**
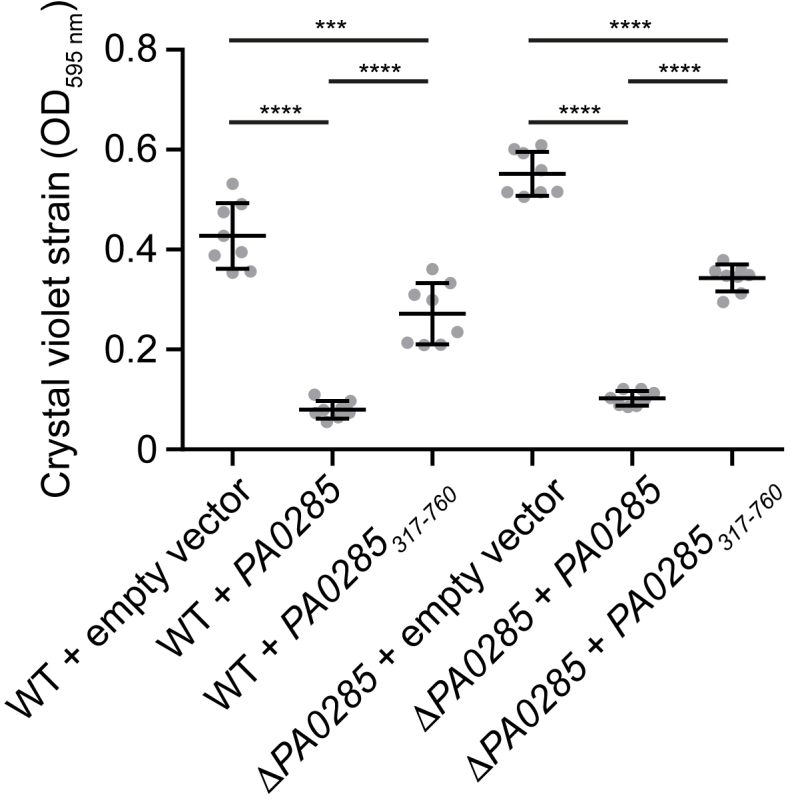
**The N-terminal portion of PA0285 is required for its function *in vivo*.** Impact of PA0285 or PA0285_317-760_ overexpression *in trans* on initial attachment of *P. aeruginosa* PAO1 or PAO1Δ*PA0285* strains after 6 h. Data represent mean ± SD of eight replicates. Unpaired two-tailed t-tests (with Welch’s correction) were used for comparison and asterisks indicate *p*-values: ∗∗∗*p* ≤ 0.001, ∗∗∗∗*p* ≤ 0.0001.

To assess a potential role of the PAS domains on the PA0285 GGDEF and EAL activities *in vitro*, we engineered constructs to express and purify variants that will include either one or two PAS domains, while still lacking the transmembrane domains (Fig. 2*A*). Whereas we were able to purify the variant containing the second PAS domain preceeding the GGDEF/EAL tandem domains (PA0285_194-760_), a full-length variant with both PAS domains (PA0285_78-760_) could not be obtained. However, we could purify a variant including the first PAS domain that lacked the EAL domain (PA0285_73-508_). However, no binding of neither FAD nor heme could be observed in MST experiments employing PA0285_194-760_ or PA0285_73-508_ (Fig. 5*D*) and DGC activity still absent for both PA0285 variants (Fig. 5*E*). Consequently, evaluation of the PDE activity of PA0285_194-760_ did not reveal any heme or FAD-dependent changes compared to PA0285_317-760_ that lacks the second PAS domain (Fig. 5*F*). Finally, we measured the impact of PA0285_317-760_ expression *in trans* on PAO1 and PAO1ΔPA0285 biofilm formation, and the downregulation of biofilm formation was less marked than when expressing the entire PA0285 (Fig. S7).

Collectively, these results suggest that the transmembrane portion of PA0285 or signal perception and transduction through one or both of the PAS domains would be required for DGC activity.

### RNA-seq and reverse transcription quantitative PCR analysis suggests that PA0285 regulates biofilm-related genes

Changes in c-di-GMP concentration, as seen in the PA0285 mutant described in this study, can indirectly or directly influence variation in gene expression. Here, we investigated the expression profile of differentially expressed genes (DEGs) in a PA0285 deletion mutant, compared to PAO1 under planktonic and biofilm growth conditions. Planktonic cells were grown for 4 h at 37 °C under shaking conditions, whereas biofilms were grown in flow-cell chambers under continuous medium flow before being harvested after 72 h. RNA extraction and transcriptomic analysis were conducted as described in the Experimental procedures section, and three technical replicates for each strain were performed. The RNA-Seq raw data were quality-checked with fastQC, filtered using SortMeRNA, trimmed using trimmomatic and mapped to the PAO1 reference genome (NC_002516.2). All DEGs with a fold change larger than two and a *p* value < 0.05 were identified using DESeq2 (https://bioconductor.org/packages/release/bioc/html/DESeq2.html) (Dataset S2). Under planktonic conditions, 53 DEGs were identified of which 27 were upregulated and 26 downregulated. In biofilm conditions, 94 genes were differentially expressed of which only 19 were upregulated and 75 downregulated. There was minimal overlap in differentially regulated genes between the lifestyle modes; and only one gene, *PA2181*, a putative ATP-dependent carboxylate-amine ligase, was downregulated in both planktonic and biofilm growth conditions.

**Figure 6:**
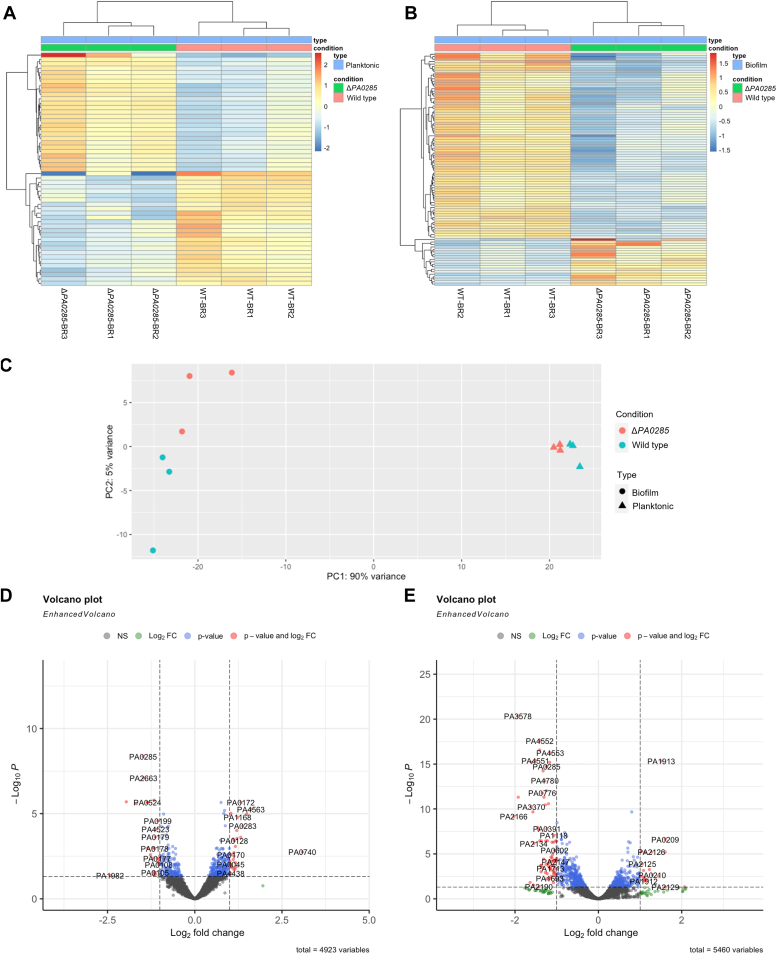
**Transcriptomic analysis of WT and Δ*PA0285* mutant in planktonic and biofilm conditions.** Transcriptomic analysis of WT and Δ*PA0285* cells in planktonic and biofilm conditions. *A*, heatmap represents genes in which the expression was upregulated or downregulated more than 2-fold in Δ*PA0285* mutant compared to WT in planktonic condition. *B*, heatmap represents genes, whereby the expression level was upregulated or downregulated more than 2-fold in Δ*PA0285* mutant compared to WT in biofilm condition. *C*, principal component analysis. *D*, *volcano plot* shows genes comparison between Δ*PA0285* and WT in planktonic condition. *E*, *volcano plot* shows genes comparison between Δ*PA0285* and WT in biofilm condition.

**Figure S8:**
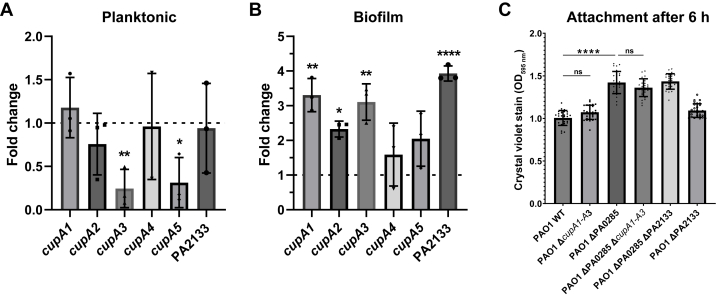
**Comparison of fimbriae CupA expression and impact on attachment.***A* and *B*, relative expression of *cupA* genes in *P. aeruginosa* PAO1ΔPA0285 compared to PAO1 WT set at 1.0 (*dotted line*), grown either, *A*, for 4 h under planktonic conditions (37 °C under shaking at 200 rpm), or *B*, for 72 h under biofilm-promoting conditions (flow cell at 25 °C). Expression levels were analyzed by RT-qPCR and normalized to the housekeeping gene *gyrA*. Data represent the means ± standard errors from three biological experiments. ∗*p* < 0.05 was considered significant using two-way ANOVA. *C*, Deletion of fimbriae genes (*cupA1-3*), *PA2133*, and *PA0285* in PAO1. Cells were grown statically at 37 °C in 96-well plates for 6 h, after which the liquid culture was removed and attached cells were stained with crystal violet. Absorbance of crystal violet was measured at 595 nm wavelength. Attachment was normalized to PAO1 WT from three biological experiments. ∗∗∗∗*p* <0.0001 based on one-way ANOVA.

The principal component analysis plot (Fig. 6*C*) shows the significant differences in transcriptomic profiles of the Δ*PA0285* mutant and WT in both planktonic and biofilm conditions. Genes whose expression level was upregulated or downregulated more than 2-fold in the Δ*PA0285* mutant compared to WT were represented by heat mapping for both planktonic (Fig. 6*A*) and biofilm conditions (Fig. 6*B*). DEGs are compared between the Δ*PA0285* mutant and WT in the volcano plots for planktonic (Fig. 6*D*) and biofilm conditions (Fig. 6*E*). Notably, genes of interest that were differentially expressed under biofilm growth conditions included the *cupA* operon, which is responsible for the functional formation of CupA fimbriae (33, 52). CupA fimbriae are cellular surface components that have previously been described as involved in attachment and initial biofilm formation (33), and their expression has been correlated to variation in c-di-GMP concentrations (53, 54, 55). In particular, the genes *cupA1* (3-fold) and *cupA2* (3-fold), directly involved in the biogenesis of CupA fimbriae, were significantly upregulated in the *PA0285* mutant compared to WT under biofilm conditions. Interestingly, PA2133, another gene belonging to the *cupA* cluster, and encoding an EAL domain-containing protein, was also identified as being upregulated by 2.7-fold. These global transcriptomic changes are also substantiated by reverse transcription quantitative PCR performed for the *cupA* genes and *PA2133* (Fig. S8). The data showed that *cupA* genes are upregulated when the Δ*PA0285* is grown under biofilm conditions (Fig. S8*B*), but not in planktonic growth (Fig. S8*A*), which is in full agreement with the RNA-Seq data. Since CupA fimbriae are known to be involved in early stages of attachment, we further explored whether introducing *cupA* gene deletion in the Δ*PA0285* mutant will abrogate the increase in attachment. As shown in Fig. S8*C*, this is not the case which suggests that other genes in addition to *cupA* may contribute the Δ*PA0285* mutant phenotype. The expression of three other GGDEF/EAL encoding genes was altered by the deletion of *PA0285* in a biofilm context, including the PAS-GGDEF/EAL domain–encoding gene *morA* (*PA4601*, upregulated 1.8-fold) (31). Furthermore, the HD-GYP motif–containing *PA4781*, a putative PDE, was downregulated by 2.7-fold (56). Other genes with significantly downregulated expression patterns included several type IV pili biogenesis genes (57), such as *fimU* (−3.0-fold), *pilV* (−2.8-fold), *pilW* (−2.7-fold), and *pilX* (−2.2-fold). An additional gene cluster, downregulated upon deletion of *PA0285* in a biofilm context, is the *mep72* operon (58). Mep72 is a metzincin protease that is normally increasingly expressed and secreted in biofilms and that affects the processing of virulence factors and flagellum-associated proteins. The *mep72* gene is significantly downregulated when *PA0285* is deleted (−3.8 fold). It is worth noting that Mep72 and BamI, whose corresponding gene is also downregulated in the *PA0285* mutant (−2.9 fold), form a complex.

Taken together, the *PA0285*-dependent changes in the transcriptome reflect its phenotypic impact on biofilm formation.

## Discussion

Many bacteria use the second messenger molecule c-di-GMP to relay various signals that result in specific phenotypic outputs. The synthesis and degradation of c-di-GMP rely on the enzymatic activity of GGDEF and EAL or HD-GYP domains, which exhibit DGC or PDE activity, respectively. Occasionally, both c-di-GMP synthesizing and degrading domains are present in a single enzyme, requiring modulation of their antagonistic activities by further accessory domains. There are insufficient studies showing that deletion of individual c-di-GMP genes often leads to a well-defined phenotype, indicating that the multiplicity of these genes does not necessarily imply redundancy (15, 17, 59). The systematic deletion of all 41 genes involved in c-di-GMP metabolism in *P. aeruginosa* PAO1 sheds light on the specific involvement of individual genes in a variety of phenotypes that are regulated by this complex and multimodal network (13). Among these, the bifunctional GGDEF/EAL domain–containing enzyme PA0285 exhibited one of the most pronounced changes in c-di-GMP levels and initial surface attachment, prompting us to further explore its role in other *Pseudomonas* species and elaborate on its biochemical properties.

Our biochemical analysis confirmed that PA0285 is a highly active PDE, hydrolyzing c-di-GMP in a manganese-dependent manner and is involved in motile-to-sessile transition-related phenotypes. The c-di-GMP hydrolytic activity of PA0285 is strictly dependent on the “ESL” motif contained in its PDE domain both *in vitro* and *in vivo*. Using HDX-MS and enzymatic assays, we could find no evidence of PDE activity modulation by any of the other domains contained in PA0285. Interestingly, we observed that GTP could inhibit the PDE activity, however, this dose-dependent inhibition was alleviated by increasing concentration of Mn^2+^, which suggests that the inhibition is likely due to Mn^2+^ scavenging by GTP. We previously observed a similar phenomenon for a manganese-dependent small alarmone hydrolase from *P. aeruginosa,* which is active against the second messengers (p)ppGpp and (p)ppApp (60).

Our phenotypic investigation of PA0285 suggested that, although PA0285 would be primarily acting as a PDE, both the EAL and DGC activities are critical to PA0285 functionality *in vivo*. This was somewhat surprising given the PA0285 GGDEF domain harbors the eponymous catalytic residues and could bind GTP *in vitro*. The reason why the GGDEF domain did not exhibit DGC activity is unclear, but could be linked to the monomeric topology of the protein under the conditions used in this study, as an active DGC enzyme relies on the formation of a homodimer effectively linking two GTP molecules together (38, 61, 62).

Consistent with the PA0285_GAAAF_ allele being unable to complement the ΔPA0285 mutant, suggesting impaired EAL activity of the enzyme, we observed a reduction of c-di-GMP hydrolysis upon GGDEF to GAAAF mutation. Reciprocal interdomain regulation between the GGDEF and EAL domains was previously reported for the *P. aeruginosa* GGDEF/EAL dual domain proteins RmcA and RbdA (28, 29), both of which link the catalytic activity of their EAL domain to the availability of GTP, likely reflecting the overall cellular nutrient level (63). In both cases, addition of GTP and subsequent binding to the GGDEF domain led to GTP-dependent allosteric increase in PDE activity (29, 63). However, using HDX-MS to ascertain whether GTP coordination by the GGDEF domain could influence ci-di-GMP binding at the EAL domain, we failed to detect conformational changes in PA0285, either by GTP in isolation or when c-di-GMP was simultaneously present. This suggests that for PA0285 the binding of c-di-GMP and GTP does not interfere with each other. We note however that the GGDEF/EAL tandem (PA0285_317-760_) and GGDEF/EAL with the second PAS domain (PA0285_194-760_) could only be obtained in pure form for our *in vitro* studies, raising the possibility that the first PAS and/or the transmembrane portion are required for DGC activity, as shown for RmcA where the first N-terminal PAS domain is particularly important in regulating the activity of its GGDEF domain (64).

Our transcriptomic analysis indicated that PA0285 may be involved in gene regulation through c-di-GMP signaling since we observed significant variation in gene expression, compared to the WT and Δ*PA0285* mutant, both in planktonic and biofilm growth conditions. The most remarkable observation was the upregulation of *cupA* genes in the Δ*PA0285* mutant, that is, with an increase in c-di-GMP levels, which corroborates the role of fimbriae in attachment and biofilm formation (33). Simultaneously, genes involved in motility are downregulated, which supports the transition between sessile and motile lifestyles (37). These results are significantly different from previous RNA-Seq data reported for a *PA0285* mutant (32). In that study, the conditions compared were planktonic *versus* autoaggregation and the genes that were the most downregulated were those involved in the production of Pf4 bacteriophage, generating the name PipA (phage inducing PDE A). In fact, comparison of RNA-Seq analysis in both studies showed very little overlap (Dataset S3), with only three upregulated genes, PA0171 (*siaB*), PA1168 (hypothetical) and PA0170 (*siaC*); and two downregulated genes, PA2664 (*fhp*) and PA0525 (*norD*), in common. These discrepancies might result from the difference in growth conditions assessed and/or parental strains used but cannot only be due to the differences in the analysis methods (see Experimental procedures). This would further indicate the complexity and sensitivity of the c-di-GMP signaling network when facing changes in environmental conditions or acting in a different genetic context.

Beyond the specific mechanism that modulates PA0285 enzymatic activity, it is also important to note that its role and function is broadly conserved across *Pseudomonas* strains and species. Notably, surface attachment and biofilm formation are controlled by a myriad of different determinants such as exopolysaccharides, extracellular appendages, and cell surface adhesins that, as a whole, lead to different phenotypic outputs among *Pseudomonas* species and even among strains or isolates of the same species. For example, PA14 and PAO1 display c-di-GMP–related phenotypic differences that might be related to their different colonialization strategies (65, 66, 67, 68). While PAO1 tends to initially attach and increase the population of surface-committed cells more rapidly, PA14 utilizes a slower approach and does not immediately attach, only slowly increasing surface coverage (69). In contrast to PA14, PAO1 utilizes a quick surge in Psl extracellular polymeric substances, the production of which is initiated by the Wsp surface–sensing system for enhanced surface attachment (70, 71).

Overall, our work implicating PA0285 at the initial attachment stage *Pseudomas* species is in agreement with published findings, and substantiates the role of PA0285-modulated c-di-GMP levels in the transition from a planktonic to sessile lifestyle. Our results further support that PA0285 is a PDE critical to the establishment of biofilm-related phenotypes and this role is universally conserved among Pseudomonads. This highlights an essential and housekeeping role for c-di-GMP in controlling biofilm processes.

## Experimental procedures

### Bacterial strains, plasmids, and growth conditions

Bacterial strains and plasmids used in this study are described in Tables S1 and S2, respectively. Oligonucleotides are listed in Table S3. *P. aeruginosa* strains (PAO1, PA14, and PAK) were grown in tryptone soy broth (Sigma), LB, or AB minimal medium supplemented with thiamine and glucose (ABTG) (15.1 mM (NH_4_)_2_SO_4_, 33.7 mM Na_2_HPO_4_ × 2 H_2_O, 22 mM KH_2_PO_4_, 50 mM NaCl, 1 mM MgCl_2_ × 6 H_2_O, 100 μM CaCl_2_ × 2 H_2_O, 1 μM FeCl_3_ × 6 H_2_O, 0.4 g of glucose per liter) and supplemented with appropriate antibiotics (carbenicillin 100 μg/ml, tetracycline 100 μg/ml or streptomycin 2 mg/ml) at 37 °C with agitation. *P. putida* (KT2440) was grown in either tryptone soy broth or LB medium at 30 °C and supplemented with appropriate antibiotic (streptomycin 2 mg/ml). *Escherichia coli* strains were grown in LB supplemented with antibiotics where appropriate (kanamycin 50 μg/ml, streptomycin 50 μg/ml, or gentamicin 50 μg/ml). IPTG was used, where stated, for *P. aeruginosa* at 250 μM and for *E. coli* at 500 μM final concentration. The *E. coli* strain DH5α was used as cloning host and BL21(DE3) was utilized for protein expression. *P. aeruginosa* chromosomal mutants were constructed as described (72). In brief, pKNG101 was transferred *via* three-partner conjugation using *E. coli* CC118 λpir as a donor and *E. coli* 1047/pRK2013 as a helper strain with counterselection by growth on 20% (w/v) sucrose.

### Crystal violet attachment assay

Attachment assays were adapted from previously published methods (73). Overnight cultures were adjusted to an *A*_600_ of 0.2, 10 μl were inoculated into 96-well plates containing 190 μl LB and subsequently incubated at 37 °C without shaking for 6 h. Wells were washed three times with distilled water and attached cell materials were stained with 0.1% (w/v) crystal violet solution (5% (v/v) methanol, 5% (v/v) isopropanol in double-distilled water) for 1 h. After staining, wells were washed three times with distilled water and crystal violet was dissolved in 20% (v/v) acetic acid solution. Absorbance of dissolved crystal violet was measured at 595 nm. Assays were performed with eight wells/strain and in three biological replicates.

### Flow cell biofilm

The flow cell system was adapted from Sternberg and Tolker-Nielsen, 2006 (74). In brief, GFP-tagged *P. aeruginosa* biofilms were grown in 40 mm × 4 mm × 1 mm three-channel flow cells with ABTG medium at 37 °C. Overnight cultures were centrifuged at 13,000*g* for 3 min and pellets were resuspended in ABTG medium and adjusted to an *A*_600_ of approx. 0.05 (inoculum). Using syringe and needle, 500 μl of inoculum was injected into each flow cell channel (three channels per strain). The flow cells were placed in an inverted position for 1 h before being reverted to an upright position and were continuously supplied with ABTG medium at the flow rate of 4 ml/h using a Cole-Parmer peristaltic pump (Cole Parmer Instrument Co). After 72 h of cultivation, the ABTG medium supply was stopped, and biofilms were analyzed.

The biofilms were observed using a LSM780 confocal laser scanning microscope (CLSM; Carl Zeiss) and seven images per channel were captured *via* a 20x/0.80 DICII objective lens and a 488 nm argon multiline laser was used to monitor the GFP-expressing bacterial cells. The excitation and emission wavelengths for GFP are 488 nm and 509 nm, respectively. The acquired images were processed using IMARIS version 9 (Bitplane AG). Experiments were performed in triplicate and representative images are shown. The acquired microscopy images were analyzed using COMSTAT2 software (https://www.comstat.dk/) to measure the three-dimensional biofilm image stack (75). The three parameters used in COMSTAT2 software to analyze the biofilm structures were biomass, mean thickness, and roughness coefficient. All COMSTAT2 parameters were fixed at default settings prior to image analysis. Experiments were performed as biological triplicates and results are shown as the mean  ±  SD

### Motility assays

Motility assays were carried out and adapted as previously described (36, 76). In brief, swimming assays were conducted on 10 g/l tryptone, 5 g/l NaCl, and 0.2% (w/v) agar (Oxoid) plates. Five hundred nanoliters of standardized overnight culture (grown in LB medium) was injected below the surface approximately into the middle of the agar and plates were incubated at 37 °C overnight (16 h). The swimming diameter was subsequently measured. Swimming assays were performed with five technical and three biological triplicates.

Twitching assays were performed on 1% (w/v) LB agar plates. Bacteria were inoculated by picking a colony with a sterile tip and stabbing it to the bottom of the plates, which were then incubated at 37 °C for 48 h. The agar was subsequently peeled off, and cells were stained with crystal violet and the twitch diameter was measured. Twitching assays were performed in three technical and three biological triplicates.

### Intracellular c-di-GMP measurement

For c-di-GMP quantification, samples were prepared as described previously (77) and analyzed by LC–MS/MS. In brief, *P. aeruginosa* strains were grown for 6 h in 10 ml of LB medium to stationary phase and cells were harvested by centrifugation. Collected cells were resuspended in 200 μl extraction solution (acetonitrile/methanol/water, 2/2/1 (v/v/v)), incubated on ice for 15 min, and heated for 10 min at 95 to 99 °C. Cells were centrifuged for 10 min at 4 °C and 20,800*g*, and supernatant fluid was collected. The extraction was repeated twice and supernatants from the three extraction steps were combined and incubated at −20 °C overnight. Extraction fluids were centrifuged again, and supernatant fluid was analyzed at the BIOLOG Life Science Institute (BIOLOG) *via* LC–MS/MS. Samples were compared to a standard curve derived from measurements of known concentrations of pure c-di-GMP to determine the concentration (in nM) of c-di-GMP in the samples, and the data were normalized to the total protein content of the sample determined by Bradford assay. For each strain, experiments were conducted in biological duplicate and LC–MS/MS measurements were taken in duplicate. Data are presented as pmol c-di-GMP/mg of total protein.

### Protein production and purification

*E. coli* BL21(DE3) cells were transformed with the respective plasmid encoding for GST-tagged PA0285 or variants thereof and proteins overproduced in LB-medium supplemented with 50 μg/ml kanamycin. Cells were grown at 37 °C under vigorous shaking to an *A*_600_ of 0.4 at which point the cultures were transferred to 20 °C. Overproduction was induced 10 min after the shift to 20 °C by addition of IPTG (final concentration 0.5 mM) and the cultures further incubated for 16 h at 20 °C. Cells were harvested by centrifugation (3500*g*, 20 min, 4 °C), resuspended in lysis buffer (20 mM Hepes-Na pH 7.5, 150 mM NaCl), and lysed with a LM10 microfluidizer (Microfluidics) at a pressure of 12,000 psi. The broken cells were centrifuged (47850*g*, 20 min, 4 °C), and the clear supernatant loaded on a GSTrap FF 5 ml column (Cytiva) equilibrated with ten column volumes (CVs) lysis buffer. After washing with ten CV of lysis buffer, proteins were eluted with five CV elution buffer (lysis buffer containing 20 mM GSH). The GST-tag was subsequently cleaved off by incubation with 100 U of bovine thrombin for 16 h at 4 °C, while at the same time dialyzing the protein solution (volume of 5 ml) in 2 l of lysis buffer at 4 °C. After completion of thrombin digestion, the protein solution was again applied to a GSTrap FF 5 ml column (Cytiva) equilibrated with ten CV lysis buffer. The flow-through was collected and concentrated (Amicon Ultracel-10K [Millipore]), and further purified by SEC on a HiLoad 26/600 Superdex 200 pg column (Cytiva) previously equilibrated with lysis buffer. PA0285-containing fractions were pooled, concentrated (Amicon Ultracel-10K [Millipore]), snap-frozen in liquid nitrogen, and stored at −80 °C. Protein concentration was determined photometrically (NanoDrop Lite, Thermo Fisher Scientific).

### Hydrogen/deuterium exchange––mass spectrometry

Nucleotide-binding to PA0285_317-760_ and PA0285_317-760, GAAAF_ was probed by HDX-MS essentially as described previously (78). Purified PA0285_317-760_ or PA0285_317-760, GAAAF_ proteins (50 μM) were supplemented with nucleotides c-di-GMP and/or GTP, dissolved in double-distilled H_2_O, with concentrations of 2.5 or 25 mM, respectively. Note that upon 10-fold dilution of the PA0285 protein samples including the nucleotides c-di-GMP or GTP, their final concentrations during HDX are 250 μM and 2.5 mM, repectively. For apo-state samples, double-distilled H_2_O was supplemented instead. The conditions and raw data of HDX-MS experiments are depicted in Dataset S1. Metal ions (MgCl_2_, MnCl_2_) were not yet present in the protein samples but added with the deuteration buffers (see below) to prohibit any enzymatic activity of PA0285 prior the HDX reaction.

HDX samples were automatically prepared by a two-arm robotic autosampler (LEAP Technologies). For both nondeuterated and deuterated HDX samples, 7.5 μl of protein solution were mixed with 67.5 μl of buffer (20 mM Hepes-Na, 150 mM NaCl, 1 mM MgCl_2_, 1 mM MnCl_2_, pH 7.5) prepared with H_2_O or D_2_O, respectively. After incubation at 25 °C for 10 s in H_2_O buffer or for 10, 100, 1000, or 10,000 s in D_2_O buffer, the H/D exchange was stopped by mixing 55 μl of the reaction with an equal volume of quench solution (400 mM KH_2_PO_4_/H_3_PO_4_, 2 M guanidine-HCl, pH 2.2) precooled at 1 °C. Ninety-five microliters of the resulting mixture were immediately injected into an ACQUITY UPLC M-class system with HDX technology (Waters) (79). Samples were flushed from the sample loop (50 μl) with water supplemented with 0.1% (v/v) formic acid at 100 μl/min flow rate and guided to a column (2 mm × 2 cm) filled with immobilized porcine pepsin for proteolytic digestion at 12 °C. The resulting peptic peptides were collected on a trap column (2 mm × 2 cm) filled with POROS 20 R2 material (Thermo Fisher Scientific), which was temperature-adjusted to 0.5 °C. After 3 min of proteolytic digestion and trapping, the trap column was placed in line with with an ACQUITY UPLC BEH C18 1.7 μm 1 × 100 mm column (Waters) kept at 0.5 °C and the peptides eluted with a gradient of water + 0.1% (v/v) formic acid (eluent A) and acetonitrile + 0.1% (v/v) formic acid (eluent B) at 30 μl/min flow rate as follows: 0 to 7 min/95-65% A, 7 to 8 min/65-15% A, 8 to 10 min/15% A. Eluting peptides were guided to and ionized with an electrospray ionization source (capillary temperature 250 °C, spray voltage 3 kV) and mass spectra acquired over a range of 50 to 2000 *m/z* on a G2-Si high definition MS (HDMS) mass spectrometer with ion mobility separation (Waters) in HDMS or enhanced HDMS mode (80, 81) for deuterated and undeuterated samples, respectively. Lock mass correction was implemented with [Glu1]-Fibrinopeptide B standard (Waters). During separation of the peptides on the C18 column, the pepsin column was washed three times by injecting 80 μl of 0.5 M guanidine hydrochloride in 4% (v/v) acetonitrile. All measurements were conducted in triplicate (independent H/D exchange reactions).

Peptides were identified with ProteinLynx Global SERVER 3.0.1 (Waters) similarly as described in (78) from the undeuterated samples acquired with enhanced HDMS employing low energy, elevated energy, and intensity thresholds of 300, 100, and 1000 counts, respectively, and matched to a database containing the amino acid sequences of PA0285_317-760_, porcine pepsin, and the reversed sequences thereof. The search parameters were as follows: peptide tolerance = automatic; fragment tolerance = automatic; min fragment ion matches per peptide = 1; min fragment ion matches per protein = 7; min peptide matches per protein = 3; maximum hits to return = 20; maximum protein mass = 250,000; primary digest reagent = nonspecific; missed cleavages = 0; false discovery rate = 100. The amount of deuterium incorporation was quantified with DynamX 3.0 (Waters; https://www.waters.com/waters/library.htm?cid=511436&lid=134832928&locale=en_US). Only peptides with a minimum intensity of 10,000 counts, a maximum length of 30 amino acids and minimal number of two product ions were considered for analysis. Mass error and retention time tolerances of 25 ppm and 0.5 min were applied. After automated data processing by DynamX, all spectra were manually inspected and curated where necessary.

### Enzymatic activity of PA0285

For DGC activity, PA0285 (10 μM) was incubated with 2.5 mM GTP and 2.5 mM of MgCl_2_, MnCl_2_, CaCl_2_, ZnCl_2_, CuCl_2_, CoCl_2_, FeSO_4_, or NiSO_4_ in lysis buffer. After incubation for 60 min at 37 °C, the reactions were quenched by addition of 150 μl chloroform followed by thorough mixing for 15 s, heating up to 95 °C for 15 s and flash-freezing in liquid nitrogen. After thawing, the samples were centrifuged (17300*g*, 10 min, 4 °C), an aliquot of the aqueous phase withdrawn and diluted 5-fold with double-distilled water. The samples were analyzed by HPLC on an Agilent 1260 Infinity system equipped with a Metrosep A Supp 5 to 150/4 column. Ten microliters of sample were injected and nucleotides eluted isocratically at a flow rate of 0.7 ml/min of 100 mM (NH_4_)_2_CO_3_ at pH 9.25 and detected at 260 nm wavelength.

For determination of PDE activity, PA0285 (2.5 μM) was incubated with 250 μM c-di-GMP and variable amounts of MnCl_2_ or MgCl_2_, as indicated in figures and text, in lysis buffer. Where indicated, FAD or heme were supplemented with 1 mM final concentration. GTP was typically employed at 2.5 mM final concentration except for Mn^2+^ scavenging experiments where GTP was present at 2.5, 10, 25, 100, 250, 1000, or 2500 μM. Assays were allowed to proceed for 60 min at 37 °C, quenched, and analyzed by HPLC as described above for DGC activity. IC_50_ values for inhibition of PA02853_17-760_ (blue) and PA0285_317-760, GAAAF_ (green) PDE activity by GTP were obtained from a fit of the enzymatic activity *versus* the log_10_ of GTP concentration. Data represent mean ± SD of n = 3 biological replicates (independent protein preparations) as per activity = min. activity + (max. activity – min. activity)/(1 + 10 ^([GTP] – log10 (IC50)^).

### Microscale thermophoresis

PA0285_317-760_ or PA0285_317-760, GAAAF_ (50 μM) were labeled with a cysteine-reactive dye (Protein Labeling Kit RED-MALEIMIDE 2nd Generation, NanoTemper Technologies) according to the manufacturer’s protocol. Unreacted dye was removed by rebuffering the labeled proteins in buffer (20 mM Hepes-Na pH 7.5, 150 mM NaCl, 1 mM MgCl_2_). MST experiments were performed on a Monolith NT.115 (NanoTemper Technologies GmbH) at 21 °C. 200 nM of labeled proteins were titrated in buffer supplemented with 0.05 mM Tween20 with compounds (GTP, c-di-GMP, FAD, heme) from approx. 15 nM to 500 μM final concentration. Three replicates (individual protein preparations) were measured at 680 nm wavelength and data processed with NanoTemper Analysis version 1.2.009 and Origin8G software suits.

### Analytical SEC

One hundred microliters of 100 μM concentrated PA0285 protein variants were applied onto a Superdex S200 10/30 Gl column (Cytiva) and eluted at 0.4 ml/min flow rate of 20 mM Hepes-Na pH 7.5, 150 mM NaCl at 4 °C. A standard curve for approximation of the molecular mass of PA0285 was obtained with a mixture of ferritin (440 kDa), aldolase (158 kDa), conalbumin (75 kDa), ovalbumin (44 kDa), and RNase A (13.7 kDa). The void volume of the column was approximated with 7.6 ml and the total column volume with 24 ml. For molecular weight determination by SEC-coupled MALS, the eluate from the SEC column was guided to a MALS and a differential refractive index detector (both Postnova Analytics), and the molecular weight calculated by combining the refraction index and MALS values by Debye fitting.

### RNA-seq analysis

For comparative transcriptomic analysis, *P. aeruginosa* cells were grown either in planktonic condition in ABTG medium for 5 h at 37 °C or under biofilm condition in flow tubes supplied with ABTG medium for 72 h at 37 °C. The total RNA was extracted from *P. aeruginosa* cells using Qiagen RNeasy Mini Kit (catalog number: 74104, Qiagen,) according to the manufacturer’s protocol. The extracted RNA samples were subjected to DNase treatment using Turbo DNA-free kit (catalog number: AM1907, Thermo Fisher Scientific), and the purified DNA-free RNA samples were subsequently subjected to ribosomal depletion using the Ribo-Zero Magnetic Kits (Illumina). The amount of RNA and DNA were measured using the Qubit RNA Assay Kits and Qubit dsDNA HS Assay Kits (Invitrogen), respectively, and the integrity of RNA was analyzed using gel electrophoresis. Complementary DNA (cDNA) were reverse transcribed using a NEBNext RNA first and second strand synthesis module (NEB). cDNA was then processed according to Illumina’s TruSeq Stranded mRNA protocol. The quantitated libraries were pooled at equimolar concentrations and sequenced on an Illumina HiSeq2500 sequencer in rapid mode at a read length of 100 bp paired ends. RNA samples were prepared in triplicate from three independent biological samples.

The total RNA samples were sequenced on Illumina HiSeq2500 sequencer at a read length of 101 bp paired end. Briefly, the quality of Illumina pair-ended reads was first assessed using FastQC v0.11 (http://www.bioinformatics.babraham.ac.uk/projects/fastqc/). RNA reads were trimmed using trimmomatic v0.38 (82) and mapped against the *P. aeruginosa* PAO1 reference genome (GenBank accession numbers: AE004091.2) using HISAT2 v2.2.1 (http://daehwankimlab.github.io/hisat2/) (83). Aligned data were sorted with SAMTools v1.13 (84) and then used as input for the feature counts function (85) from the Rsubread v2.4.3 package (86) to generate a matrix of annotated genes with their corresponding raw counts. An average of 95.46% reads were successfully mapped to the reference genome. The count data were then analyzed for differential gene expression levels and statistical significance using DESeq2 v1.30.1 (87). Genes with absolute value of Log2 fold change (Log2FC) ≥1 and adjusted *p* value ≤ 0.05 were considered as DEGs in comparative analysis. RNA raw reads have been deposited in the Gene Expression Omnibus database under accession GSE223663. Gene Ontology and Kyoto Encyclopedia of Genes and Genomes annotation was downloaded from The *Pseudomonas* Genome Database (https://www.pseudomonas.com/) (88). Heatmaps were plotted with ComplexHeatmap (89). Sample distances were calculated with DEseq2 VST normalized count table and plotted with pheatmap package (https://cran.r-project.org/web/packages/pheatmap/index.html). Volcano plots were plotted with EnhancedVolcano (https://github.com/kevinblighe/EnhancedVolcano).

The comparison between our analysis and the PipA analysis considered the following adjustments. The PipA differential expressed gene list was extracted from the Table S1 from the previous paper (32). It is reported in this previous study that there are 1388 DEGs with a 2-fold cut-off and *p* < 0.01 and among these, 361 genes were upregulated or down regulated over 4-fold. This analysis used a combination of gene symbols and locus identifiers (IDs), while we only use locus IDs. To make a consistent comparison with our result we did the following: first, the gene symbols of PipA dataset were converted to locus IDs. As some gene symbols match to multiple locus IDs, this ended up with 1347 genes (upregulated 507 genes and down-regulated 844 genes) for PipA dataset. There were two genes, with gene symbol as *cynT* and *lepA_2*, which could not be converted to locus IDs. Second, we changed the cut-off for adjusted *p* value from 0.05 to 0.01 for our dataset, which cut down the total of 53 (27 upregulated genes and 26 downregulated genes) to 41 (21 upregulated genes and 20 downregulated genes). We also checked the top upregulated and downregulated genes. However, the top five upregulated and downregulated genes in the PipA dataset do not appear in our results. The top five upregulated genes (PA3234, PA1202, PA4139, PA1168, PA0887) in the PipA dataset all have more than 20-fold changes and the top five downregulated genes (PA0285, PA0723, PA0721, PA0720, PA0716.2) have more than 600-fold changes. Most of these are linked to bacteriophage Pf4. However, in our dataset, we did not observe these differences.

### Reverse transcription quantitative PCR

For reverse transcription quantitative PCR, cells were grown in ABTG in either planktonic condition for 4 h at 37 °C or under biofilm condition in 40 mm × 4 mm × 1 mm three-channel flow cells (74). Total RNA was extracted from the flow cells using RNAprotect bacteria reagent and RNeasy Mini Kit (both Qiagen) according to manufacturer’s protocols. Samples were treated with Turbo-DNA free kit (Thermo Fisher Scientific) to remove contaminating DNA and cleaned using RNA Clean & Concentrator (Zymo Research) as per the manufacturer’s instructions. RNA and DNA concentrations were quantified with Qubit RNA broad range assay kit and Qubit dsDNA high sensitivity on Qubit 2.0 Fluorometer (Invitrogen). RNA integrity was also checked using RNA ScreenTape on 4200 TapeStation system (Agilent) for RNA integrity. RNA samples were diluted to match the sample with the lowest RNA concentration. Equal volumes for all samples were then converted into cDNA with random hexamers as primers using SuperScript IV First-Stand synthesis system (Thermo Fisher Scientific). A housekeeping gene, *gyrA*, was included for normalization. All qPCR primers were designed using PrimerBLAST and assays were carried out using the StepOnePlus real-time PCR system in a 96-well plate format. Amplification of cDNA was performed in 20 μl reactions of PowerUp SYBR green mastermix (Thermo Fisher Scientific), forward and reverse primers at a final concentration of 300 nM and 1 μl of cDNA sample. The PCR conditions were 50 °C for 2 min, 95 °C for 2 min, 40 cycles of 95 °C for 15 s, and 60 °C for 1 min, with a final melting curve using these parameters: 95 °C for 15 s, 60 °C for 1 min, then a slow ramp (0.3 °C/s) to 95 °C.

## Data availability

Source Data are provided with this article. RNA-Seq data are available from the Gene Expression Omnibus repository database under the ID GSE223663. All other requests for data and materials should be addressed to W. S. and A. F.

## Supporting information

This article contains Supporting information (43, 47, 49, 72, 73).

## Conflict of interest

The authors declare that they have no conflicts of interest with the contents of this article.
