# Supplementary material for: The dual GGDEF/EAL domain enzyme PA0285 is a Pseudomonas species housekeeping phosphodiesterase regulating early attachment and biofilm architecture
Source: J Biol Chem. 2024 Jan 16;300(2):105659. doi: 10.1016/j.jbc.2024.105659 (PMC10874727; doi:10.1016/j.jbc.2024.105659)
Supplement: Supporting information [file mmc4.docx]

**Supplemental data set legends**

**Supplemental Dataset 1.** The spreadsheet provides a summary of the conditions used for HDX-MS analyses of PA0285 and a full list of the peptides obtained in the experiments.

**Supplemental Dataset 2.** The spreadsheet provides a list of differentially regulated genes of *P. aeruginosa* PAO∆*PA0285* compared to WT during planktonic and biofilm growth conditions.

**Supplemental Dataset 3.** The spreadsheet provides a comparison between our dataset (supplemental dataset 2) with the PipA dataset (1) of differentially regulated genes in the *P. aeruginosa* ∆*PA0285* mutant compared to WT during planktonic growth conditions.

**Supplemental figures legends**

**Figure S1. Biofilm characteristics of PAO1 mutant strains. A.** Micrographs of *P. aeruginosa* PAO1 WT, the *PA0285* deletion and *PA0285* deletion strains complemented with either empty vector or *PA0285*. Scale bar, 50 µm. GFP-tagged PAO1 WT and mutant strains were grown for 72 h in biofilm flow cell chambers under continuous feeding of ABTG medium. Confocal scanning laser microscopy images were taken (three channels: seven images per channel). A representative image for each strain is shown. **B.** Thickness, biomass and roughness coefficient (indicator of biofilm heterogeneity) of *P. aeruginosa* PAO1 and mutant strain from **A** were analyzed using biofilm quantification program COMSTAT. Data represent mean ± SD of three biological replicates. Unpaired two-tailed t-tests (with Welch’s correction) were used to compare the mutant strains with WT. Asterisks indicate *p*-values: * *p* ≤ 0.05; ns, not significant. Determined *p*-values are: thickness: 0.3478 (WT vs. Δ*PA0285*), 0.2944 (WT vs. Δ*PA0285* + empty vector), 0.5767 (WT vs. Δ*PA0285* + *PA0285*); biomass: 0.5718 (WT vs. Δ*PA0285*), 0.3950 (WT vs. Δ*PA0285* + empty vector), 0.8017 (WT vs. Δ*PA0285* + *PA0285*); roughness coefficient: 0.0286 (WT vs. Δ*PA0285*), 0.0329 (WT vs. Δ*PA0285* + empty vector), 0.1923 (WT vs. Δ*PA0285* + *PA0285*).

**Figure S2. Conservation of *PA0285* orthologs among *Pseudomonas* species. A-F.** Neighboring genes of *PA0285* orthologs in **A.** *P. aeruginosa* PAO1, **B.** *P. aeruginosa* UCBPP-PA14, **C.** *P. aeruginosa* PAK, **D.** *P. syringae pv. tomato* DC3000, **E.** *P. protegens* Pf-5, and **F.** *P. putida* KT2440 retrieved from the *Pseudomonas* Genome Database (2). Numbers in parenthesis denote the base pairs of the displayed genome sections. Note that in *P. syringae pv. tomato* DC3000, *P. protegens* Pf-5 and *P. putida* KT2440 the *PA0285* ortholog-enframing genes are inverted. Note that the orientation was inverted in panels D-F for better comparability.

**Figure S3. The GGDEF/EAL tandem domains of PA0285_317-760_ do not exhibit DGC activity *in vitro*. A-D.** Representative UV traces of enzymatic reactions probing DGC activity of 2.5 µM **A.** PA0285_317-760_, **B.** PA0285_317-760, ASA_, **C.** PA0285_353-508_, or **D.** PA0285_194-760_ in presence of 2.5 mM GTP substrate and 2.5 mM of various divalent metal ion cofactors provided as MgCl_2_, MnCl_2_, CaCl_2_, ZnCl_2_, CuCl_2_, CoCl_2_, FeSO_4_, or NiSO_4_. The reactions were allowed to proceed for 60 min at 37°C prior quenching and analysis of nucleotide content by HPLC analysis. A mixture of GTP and c-di-GMP (250 µM each) served as standard for identification based on retention time. Panels **E-H** display representative UV traces of enzymatic reactions for DGC activity employing 2.5 µM of GST-tagged proteins, i.e., **E.** GST-PA0285_317-760_, **G.** GST-PA0285_317-760, ASA_, **G.** GST-PA0285_353-508_, or **H.** GST-PA0285_194-760_ similarly. Note that both tagless and GST-tagged PA0285_353-508_ (panels C and G, respectively) showed elevated GDP probably attributable to an unknown GTPase contaminant in our preparations of this particular variant. Experiments done in panels A-B, D-F and H employed the same reagents and standards (black trace displays identical data in these panels).

**Figure S4. HDX-MS analysis of the PA0285_317-760, GAAAF_ variant. A, C.** The difference in HDX of between **A.** PA0285_317-760, GAAAF_ and PA0285_317-760_ in the apo states, and **C.** PA0285_317-760, GAAAF_ in presence of GTP versus apo state, projected on the amino acid sequence. The predicted secondary structure is indicated above both plots (red boxes, α-helices; black arrows, β-strands). HDX is given per residue (**Supplemental dataset 1**). **B, D.** Progression of HDX over time displayed for six selected representative peptides for **B.** the apo states of PA0285_317-760, GAAAF_ and PA0285_317-760_, and **D.** PA0285_317-760, GAAAF_ in presence of GTP versus apo state. Data represent mean ± SD of three technical replicates (independent HDX reactions).

**Figure S5. Mn^2+^ scavenging by GTP impedes PA0285 PDE activity *in vitro*. A, B.** Representative UV traces of enzymatic reactions containing 2.5 µM of **A.** PA0285_317-760_, or **B.** PA0285_317-760, GAAAF_, probing EAL activity in presence of c-di-GMP substrate, 250 or 2,500 µM Mn^2+^ ion cofactor and GTP at 0, 100 or 1,000 µM final concentration. The reactions were incubated for 60 min at 37°C prior quenching and analysis of nucleotide content by HPLC analysis. A mixture of pGpG and c-di-GMP (250 µM each) served as standard for identification based on retention time. **C.** Quantification of PDE activity of reactions shown in **A** and **B.** Data represent mean ± SD of three biological replicates (independent protein preparations). **D-I.** IC_50_ values for inhibition of PA0285_317-760_ (blue) and PA0285_317-760, GAAAF_ (green) PDE activity by GTP were obtained from a fit of the enzymatic activity versus the log_10_ of GTP concentration. Reactions contained 2.5 µM PA0285, 250 µM c-di-GMP, MnCl_2_ at 10, 25, 100, 250, 1,000 or 2,500 µM, and GTP was supplemented at 0, 2.5, 10, 25, 100, 250, 1,000 or 2,500 µM final concentration. Assays were allowed to proceed for 60 min at 37°C prior quenching and HPLC analysis. Data represent mean ± SD of three biological replicates (independent protein preparations).

**Figure S6. Potential dimerization and ligand binding by the PA0285 PAS domains. A.** AlphaFold-generated model (3) of the PA0285 residues 1-196 (TM and PAS1 domains) is shown inserted into a simplified lipid bilayer. Hydrophobic residues potentially involved in dimerization are indicated as red sticks. **B.** AlphaFold structural model of the first PAS domain (residues 79-207) of PA0285 (AF-Q9I6K5-F1-model_v4 (3)), the crystal structure of the heme PAS sensor domain (residues 20-132) from *Escherichia coli* DOS (PDB-ID 1V9Y (4) and their superposition. The heme-ligated histidine residue of *E. coli* DOS and its potential counterpart in PA0285 PAS1 are indicated. **C.** AlphaFold structural model of the second PAS domain (residues 204-329) of PA0285 (AF-Q9I6K5-F1-model_v4 (3,5), the crystal structure of the FAD-containing redox sensor PAS domain (residues 22-140) from *Azotobacter vinelandii* NifL (PDB-ID 2GJ3 (6) and their superposition. The tryptophane and asparagine residues conferring FAD coordination by *A. vinelandii* NifL and their counterparts in PA0285 are indicated.

**Figure S7.** **The N-terminal portion of PA0285 is required for its function *in vivo*.** Impact of PA0285 or PA0285_317-760_ overexpression *in trans* on initial attachment of *P. aeruginosa* PAO1 or PAO1Δ*PA0285* strains after 6 h. Data represent mean ± SD of eight replicates. Unpaired two-tailed t-tests (with Welch’s correction) were used for comparison and asterisks indicate *p*-values: *** *p* ≤ 0.001, **** *p* ≤ 0.0001.

**Figure S8.** **Comparison of fimbriae CupA expression and impact on attachment. A-B.** Relative expression of *cupA* genes in *P. aeruginosa* PAO1∆PA0285 compared to PAO1 WT set at 1.0 (dotted line), grown either, **A.** for 4 h under planktonic conditions (37°C under shaking at 200 rpm), or **B.** for 72 h under biofilm-promoting conditions (flow cell at 25°C). Expression levels were analyzed by RT-qPCR and normalized to the housekeeping gene *gyrA*. Data represent the means ± standard errors from three biological experiments. **p* < 0.05 was considered significant using two-way ANOVA. **C.** Deletion of fimbriae genes (*cupA1-3*), *PA2133*, and *PA0285* in PAO1. Cells were grown statically at 37°C in 96-well plates for 6 h, after which the liquid culture was removed and attached cells were stained with crystal violet. Absorbance of crystal violet was measured at 595 nm wavelength. Attachment was normalized to PAO1 WT from three biological experiments. **** *p* <0.0001 based on one-way ANOVA.

**References**

1. Cai, Y. M., Yu, K. W., Liu, J. H., Cai, Z., Zhou, Z. H., Liu, Y., Wang, T. F., and Yang, L. (2022) The c-di-GMP Phosphodiesterase PipA (PA0285) Regulates Autoaggregation and Pf4 Bacteriophage Production in Pseudomonas aeruginosa PAO1. *Appl Environ Microbiol* **88**, e0003922

2. Winsor, G. L., Griffiths, E. J., Lo, R., Dhillon, B. K., Shay, J. A., and Brinkman, F. S. (2016) Enhanced annotations and features for comparing thousands of Pseudomonas genomes in the Pseudomonas genome database. *Nucleic Acids Res* **44**, D646-653

3. Jumper, J., Evans, R., Pritzel, A., Green, T., Figurnov, M., Ronneberger, O., Tunyasuvunakool, K., Bates, R., Zidek, A., Potapenko, A., Bridgland, A., Meyer, C., Kohl, S. A. A., Ballard, A. J., Cowie, A., Romera-Paredes, B., Nikolov, S., Jain, R., Adler, J., Back, T., Petersen, S., Reiman, D., Clancy, E., Zielinski, M., Steinegger, M., Pacholska, M., Berghammer, T., Bodenstein, S., Silver, D., Vinyals, O., Senior, A. W., Kavukcuoglu, K., Kohli, P., and Hassabis, D. (2021) Highly accurate protein structure prediction with AlphaFold. *Nature* **596**, 583-589

4. Kurokawa, H., Lee, D. S., Watanabe, M., Sagami, I., Mikami, B., Raman, C. S., and Shimizu, T. (2004) A redox-controlled molecular switch revealed by the crystal structure of a bacterial heme PAS sensor. *J Biol Chem* **279**, 20186-20193

5. Varadi, M., Anyango, S., Deshpande, M., Nair, S., Natassia, C., Yordanova, G., Yuan, D., Stroe, O., Wood, G., Laydon, A., Zidek, A., Green, T., Tunyasuvunakool, K., Petersen, S., Jumper, J., Clancy, E., Green, R., Vora, A., Lutfi, M., Figurnov, M., Cowie, A., Hobbs, N., Kohli, P., Kleywegt, G., Birney, E., Hassabis, D., and Velankar, S. (2022) AlphaFold Protein Structure Database: massively expanding the structural coverage of protein-sequence space with high-accuracy models. *Nucleic Acids Res* **50**, D439-D444

6. Key, J., Hefti, M., Purcell, E. B., and Moffat, K. (2007) Structure of the redox sensor domain of Azotobacter vinelandii NifL at atomic resolution: signaling, dimerization, and mechanism. *Biochemistry* **46**, 3614-3623
