# Supplementary material for: The dual GGDEF/EAL domain enzyme PA0285 is a Pseudomonas species housekeeping phosphodiesterase regulating early attachment and biofilm architecture
Source: J Biol Chem. 2024 Jan 16;300(2):105659. doi: 10.1016/j.jbc.2024.105659 (PMC10874727; doi:10.1016/j.jbc.2024.105659)
Supplement: Tables S1-S3 [file mmc5.docx]

**Table S1. Strains used in this study.**

| Strain | Relevant features | Reference |
| --- | --- | --- |
| DH5α | Strain used for cloning and maintenance of plasmids. F^–^*endA1* *glnV44* *thi*^-^. *recA1* *relA1 gyrA96* *deoR nupG purB20* φ80d*lacZ*ΔM15 Δ(*lacZYA*-*argF*)U169, *hsdR17*(r*_K_*^-^m*_K_*^+^ ),λ^-^. | Invitrogen |
| BL21(DE3) | Strain used for protein expression systems. F^–^ *ompT gal dcm lon hsdS_B_*(r_B_–m_B_–) λ(DE3 [*lacI* l*acUV5*- *T7p07* *ind1 sam7 nin5*]) [*malB*^+^]_K-12_(λS). | Despoina Mavridou |
| CC118λpir | Strain used for pKNG101 maintaining . Δ(*ara-leu*) *araD* Δ*lacX74* *galE* *galK-phoA20 thi-1 rpsE rpoB argE* (ApR) *recA1* Rfr λ*pir*. | (74) |
| PAO1 | Wild-type *P. aeruginosa* strain, parental used for all experiments, Lausanne subline | Dieter Haas |
| PAO1 ∆*PA0285* | Deletion of *PA0285* in *P. aeruginosa* PAO1 | This study |
| PAK | Wild-type *P. aeruginosa* PAK strain | Laboratory collection |
| PAK ∆*PA0285* | Deletion of *P. aeruginosa* PAK *PA0285* homologue (PAK_00495) | This study |
| PA14 | Wild-type *P. aeruginosa* PA14 strain | Laboratory collection |
| PA14 ∆*PA0285* | Deletion of *P. aeruginosa* PA14 *PA0285* homologue (PA14_03720) | This study |
| KT2440 | Wild-type *P. putida* KT2440 strain | Laboratory collection |
| KT2440 ∆*PA0285* | Deletion of *P. putida* KT2440 *PA0285* homologue (*PP_0218*) | This study |
| PAO1  ∆*cupA1-A3* | Deletion of *cupA1-A3* in *P. aeruginosa* PAO1 | This study |
| PAO1 ∆*PA0285*  ∆*cupA1-A3* | Deletion of *cupA1-A3* in *P. aeruginosa* PAO1∆*PA0285* | This study |
| PAO1  ∆*PA2133* | Deletion of *PA2133* in *P. aeruginosa* PAO1 | This study |
| PAO1 ∆*PA0285*  ∆*PA2133* | Deletion of *PA2133* in *P. aeruginosa* PAO1∆*PA0285* | This study |

**Table S2. Plasmids used in this study.**

| Plasmid | Characteristics | Source |
| --- | --- | --- |
| Cloning vectors | | |
| pCRTM-Blunt II-TOPOTM | Sub-cloning vector for constructs synthesized by KOD PCR, kanamycin resistance | Invitrogen |
| pRK2013 | Self-transmissible helper plasmid for three-partner conjugations, kanamycin resistance | (75) |
| Chromosomal mutagenesis vectors | | |
| pKNG101 | Non-replicative suicide vector for *P. aeruginosa* chromosome mutagenesis, ori6K, mobRK2, *sacB* gene for sucrose sensitivity, streptomycin resistance | (76) |
| pKNG101: ∆PA0285 | Suicide vector for deletion of *PA0285* from *P. aeruginosa* species, streptomycin resistance | This study |
| pKNG101: ∆PP0218 | Suicide vector for deletion of *PA0285* homologue PP0218 from *P. putida*, streptomycin resistance | This study |
| Expression vectors for *Pseudomonas* species | | |
| pBBR-MCS-4 | Broad host range expression vector, constitutive pLAC promoter, pBBR1 ori, beta-lactam resistance | This study |
| pBBR-MCS-4: PA0285 | Constitutive expression of *PA0285*, beta-lactam resistance | This study |
| pBBR-MCS-4: PA0285_ASA_ | Constitutive expression of PDE domain catalytic mutant PA0285 (ESL to ASA), beta-lactam resistance | This study |
| pBBR-MCS-4: PA0285_GAAAF_ | Constitutive expression of DGC domain catalytic mutant PA0285 (GGDEF to GAAAF), beta-lactam resistance | This study |
| pBBR-MCS-4:  PA0285_317-760_ | Constitutive expression of trunctated (317-760) PA0285, beta-lactam resistance | This study |
| Expression vectors for *E. coli* | | |
| pet41a: PA0285_317-760_ | Expression of truncated (317-760) PA0285 with N-terminal GST, kanamycin resistance | This study |
| pet41a PA0285_317-760, ASA_ | Expression of truncated (317-760) PA0285 with N-terminal GST and PDE domain catalytic mutation (ESL to ASA), kanamycin resistance | This study |
| pet41a PA0285_317-760, GAAAF_ | Expression of truncated (317-760) PA0285 with N-terminal GST and DGC domain catalytic mutation (GGDEF to GAAAF), kanamycin resistance | This study |
| pet41a: PA0285_194-760_ | Expression of truncated (194-760) PA0285 with N-terminal GST, kanamycin resistance | This study |
| pet41a: PA0285_353-508_ | Expression of truncated (353-508) PA0285 with N-terminal GST, kanamycin resistance | This study |
| pet41a: PA0285_73-508_ | Expression of truncated (73-508) PA0285 with N-terminal GST, kanamycin resistance | This study |

**Table S3. Oligonucleotides used in this study.**

| Construct | Code | Sequence |
| --- | --- | --- |
| pKNG101: ∆PA0285 | OAL5476 | ACGGTGATCTCGACGGCG |
|  | OAL5477 | CGCCGCAAGCTGGCCATCGACGACTTC |
|  | OAL5478 | GATGGCCAGCTTGCGGCGGAACTGGCG |
|  | OAL5479 | ACGGTGGATAGCGGTCCA |
|  | OAL5480 | CATTCGCGGCCGACAGCT |
|  | OAL5481 | CACGGCCCAGACGGTCAG |
| pKNG101: ∆PP0218 | OAL5934 | AGAAGTGCTTGAGCGCGCC |
|  | OAL5935 | GTGCAGCACATGGCCGATGAAATTGAGCAGGC |
|  | OAL5936 | ATCGGCCATGTGCTGCACGACACCGGC |
|  | OAL5937 | CTTGATCAACTCGTCGGCGATGG |
|  | OAL5938 | TGCCATAGATGCGCAGGG |
|  | OAL5939 | GATTCCATTCACCCGCCTG |
| pBBR-MCS-4: PA0285 | OAL5570 | GTACGGGATCCTAACAGGAGGAATTAACC ATGAGCCCCCGCCTGAGT |
|  | OAL5572 | GACTGGTACC TCAGTCTTCCGGCAGCGCC |
| pBBR-MCS-4: PA0285_ASA_ | OAL5852 | CTGTCGCGACTGGGCGCCGCCGCATTCGCCATCCTCGTC |
|  | OAL5853 | GACGAGGATGGCGAATGCGGCGGCGCCCAGTCGCGACAG |
| pBBR-MCS-4: PA0285_GAAAF_ | OAL5854 | ATCGTCGGCGTCGCGTCGGCGGTGCGCTGGCAGC |
|  | OAL5855 | GCTGCCAGCGCACCGCCGACGCGACGCCGACGAT |
| pBBR-MCS-4:  PA0285_317-760_ | OAL5879 | GACTGGATCCAAGCGCTCGGAGAACGAACTGGA |
|  | OAL5569 | GACTGAGCTCTCAGTCTTCCGGCAGCGCC |
| pet41a: PA0285_317-760_ | OAL5879 | GACTGGATCCAAGCGCTCGGAGAACGAACTGGA |
|  | OAL5569 | GACTGAGCTCTCAGTCTTCCGGCAGCGCC |
| pet41a PA0285_317-760, ASA_ | OAL5854 | ATCGTCGGCGTCGCGTCGGCGGTGCGCTGGCAGC |
|  | OAL5855 | GCTGCCAGCGCACCGCCGACGCGACGCCGACGAT |
| pet41a PA0285_317-760, GAAAF_ | OAL5852 | CTGTCGCGACTGGGCGCCGCCGCATTCGCCATCCTCGTC |
|  | OAL5853 | GACGAGGATGGCGAATGCGGCGGCGCCCAGTCGCGACAG |
| pet41a: PA0285_194-760_ | OAL6418 | GACTGGATCC GGCAGCGAGCAGCGCCTG |
|  | OAL5569 | GACTGAGCTCTCAGTCTTCCGGCAGCGCC |
| pet41a: PA0285_353-508_ | OAL6665 | ATATGAGCTCCTGGAGAACGGCAAGGACCGG |
|  | OAL6664 | ATATAAGCTTGGCCGACTCCACCTGGACATG |
| pet41a: PA0285_73-508_ | OAL6666 | ATATGAGCTCTACGACGAACTGCACGGCAGC |
|  | OAL6664 | ATATAAGCTTGGCCGACTCCACCTGGACATG |
| pKNG101∆*cupA1-cupA3* | ∆*cupA1-cupA3*_P1 | ACGGATCCCCGGGTACCATAGGAAATTGCCCGGGAAAAGT |
|  | ∆*cupA1-cupA3*_P2 | AGGTTGGAGTCGGTGGTGGCGTGATAGTGTTTGCCGCCAT |
|  | ∆*cupA1-cupA3*_P3 | ATGGCGGCAAACACTATCACGCCACCACCGACTCCAAC |
|  | ∆*cupA1-cupA3*_P4 | AAAGACTCTAGACCTAGGCCTGGCGCTCCTTCCAGATC |
|  | ∆*cupA1-cupA3*_P5 | GCCCACTTGCAACGAGAG |
|  | ∆*cupA1-cupA3*_P6 | GATAGCGTTTCAGGGTCACG |

**Table S3 (continued). Oligonucleotides used in this study.**

| Construct | Code | Sequence | |
| --- | --- | --- | --- |
| pKNG101∆*PA2133* | ∆*PA2133*_P1 | AACCGGATACCGCGCTGGC | |
|  | ∆*PA2133*_P2 | TCACCCCTGACCGTTCACTGTAGAGC | |
|  | ∆*PA2133*_P3 | GTGAACGGTCAGGGGTGACCGGGGAGG | |
|  | ∆*PA2133*_P4 | CCTGGGTGAACAGTTCCACG | |
|  | ∆*PA2133*_P5 | ACGGCGGGGCGAGTCGAGG | |
|  | ∆*PA2133*_P6 | GCCGAGCGTCAGCAGGATG | |
| qPCR primers | | | |
| Gene | Forward | | Reverse |
| *cupA1* | GCAGTGGTATTGGCCTTTGC | | CAGGTCTGGTCGGTCACTTC |
| *cupA2* | CTCAACGTCCTGCAGATCCC | | GGTAGAACAGCTTCAGGCGA |
| *cupA3* | GCGGTTACGGCAACCTCTAT | | TGCCCCCAGGTATTGGAGTA |
| *cupA4* | ACCCTGAAACGCTATCCCAC | | GCTCTGGCACTTGAAGGTGA |
| *cupA5* | CGAATCGCTGTACTGGCTGA | | AGCACCTTCATCTGGGTTCG |
| *PA2133* | CCGTCCCGATGTGATCAAGA | | TCCTCCTCGAGACCTTCCAG |
| *gyrA* | TGTGCTTTATGCCATGAGCGA | | TCCACCGAACCGAAGTTGC |
